# Patterns of Growth in Childhood in Relation to Adult Schooling Attainment and Intelligence Quotient in 6 Birth Cohorts in Low- and Middle-Income Countries: Evidence from the Consortium of Health-Oriented Research in Transitioning Societies (COHORTS)

**DOI:** 10.1093/jn/nxab096

**Published:** 2021-05-12

**Authors:** Natalia E Poveda, Fernando P Hartwig, Cesar G Victora, Linda S Adair, Fernando C Barros, Santosh K Bhargava, Bernardo L Horta, Nanette R Lee, Reynaldo Martorell, Mónica Mazariegos, Ana M B Menezes, Shane A Norris, Linda M Richter, Harshpal Singh Sachdev, Alan Stein, Fernando C Wehrmeister, Aryeh D Stein, COHORTS Group, Natalia P Lima, Helen Goncalves, Bruna Goncalves C da Silva, Paula D de Oliveira, Joseph Murray, Feziwe Mpondo, Lukhanyo Nyati, Caroline H D Fall, Clive Osmond, Lakshmy Ramakrishnan, Sikha Sinha, Bhaskar Singh, Manuel Ramirez-Zea, Maria F Kroker-Lobos, Isabelita Bas, Sonny Agustin Bechayda, Delia Carba, Tita Lorna Perez, Charlotte Wray, Gaia Scerif

**Affiliations:** Nutrition and Health Sciences, Laney Graduate School, Emory University, Atlanta, GA, USA; Postgraduate Program in Epidemiology, Federal University of Pelotas, Pelotas, Brazil; Medical Research Council Integrative Epidemiology Unit, University of Bristol, Bristol, United Kingdom; Postgraduate Program in Epidemiology, Federal University of Pelotas, Pelotas, Brazil; Department of Nutrition, Gillings School of Global Public Health, University of North Carolina at Chapel Hill, Chapel Hill, NC, USA; Postgraduate Program in Health and Behaviour, Universidade Católica de Pelotas, Pelotas, Brazil; Consultant Pediatrician and Founder New Delhi Birth Cohort, New Delhi, India; Postgraduate Program in Epidemiology, Federal University of Pelotas, Pelotas, Brazil; USC-Office of Population Studies Foundation, Inc, University of San Carlos – TC, Talamban, Cebu City, Cebu, Philippines; Nutrition and Health Sciences, Laney Graduate School, Emory University, Atlanta, GA, USA; Hubert Department of Global Health, Rollins School of Public Health, Emory University, Atlanta GA, USA; INCAP Research Center for the Prevention of Chronic Diseases (CIIPEC), Institute of Nutrition of Central America and Panama (INCAP), Guatemala City, Guatemala; Postgraduate Program in Epidemiology, Federal University of Pelotas, Pelotas, Brazil; SAMRC Developmental Pathways for Health Research Unit, University of the Witwatersrand, Johannesburg, South Africa; Global Health Research Institute, School of Human Development and Health & NIHR Southampton Biomedical Research Centre, University of Southampton, United Kingdom; DSI-NRF Centre of Excellence in Human Development, University of the Witwatersrand, Johannesburg, South Africa; Senior Consultant Pediatrics and Clinical Epidemiology, Sitaram Bhartia Institute of Science and Research, New Delhi, India; Department of Psychiatr, University of Oxford, Oxford, United Kingdom; MRC/Wits Rural Public Health and Health Transitions Research Unit (Agincourt), School of Public Health, Faculty of Health Sciences, University of the Witwatersrand, Johannesburg, South Africa; Postgraduate Program in Epidemiology, Federal University of Pelotas, Pelotas, Brazil; Nutrition and Health Sciences, Laney Graduate School, Emory University, Atlanta, GA, USA; Hubert Department of Global Health, Rollins School of Public Health, Emory University, Atlanta GA, USA

**Keywords:** intelligence quotient, schooling attainment, growth, conditional height, conditional relative weight, cohort studies

## Abstract

**Background:**

Growth faltering has been associated with poor intellectual performance. The relative strengths of associations between growth in early and in later childhood remain underexplored.

**Objectives:**

We examined the association between growth in childhood and adult human capital in 5 low- and middle-income countries (LMICs).

**Methods:**

We analyzed data from 9503 participants in 6 prospective birth cohorts from 5 LMICs (Brazil, Guatemala, India, the Philippines, and South Africa). We used linear and quasi-Poisson regression models to assess the associations between measures of height and relative weight at 4 age intervals [birth, age ∼2 y, midchildhood (MC), adulthood] and 2 dimensions of adult human capital [schooling attainment and Intelligence Quotient (IQ)].

**Results:**

Meta-analysis of site- and sex-specific estimates showed statistically significant associations between size at birth and height at ∼2 y and the 2 outcomes (*P* < 0.001). Weight and length at birth and linear growth from birth to ∼2 y of age (1 *z*-score difference) were positively associated with schooling attainment (β: 0.13; 95% CI: 0.08, 0.19, β: 0.17; 95% CI: 0.07, 0.32, and β: 0.25, 95% CI: 0.10, 0.40, respectively) and adult IQ (β: 0.74, 95% CI: 0.35, 1.14, β: 0.73, 95% CI: 0.35, 1.10, and β: 1.52, 95% CI: 0.96, 2.08, respectively). Linear growth from age 2 y to MC and from MC to adulthood was not associated with higher school attainment or IQ. Change in relative weight in early childhood, MC, and adulthood was not associated with either outcome.

**Conclusions:**

Linear growth in the first 1000 d is a predictor of schooling attainment and IQ in adulthood in LMICs. Linear growth in later periods was not associated with either of these outcomes. Changes in relative weight across the life course were not associated with schooling and IQ in adulthood.

 

## Introduction

Growth faltering remains widespread in low- and middle-income countries (LMIC), with 96.8 million children aged <5 y being stunted (1). The period between conception and the second birthday is critical for human growth (2) as well as for development of brain structure, architecture, and function (3). In early childhood, sensory and motor functions develop, followed by acquisition of basic language skills and spatial attention (3). Evidence from birth cohort studies indicates that linear growth in the first 24 mo after birth (whether represented as attained length/height or as change in length/height) is among the strongest predictors of school attainment (4, 5), being positively associated with more years of schooling, lower probability of repeating a grade, younger age of enrollment in school, and better verbal and nonverbal skills (4, 6–8). Further, lower schooling attainment is related to future disadvantages such as lower wages (7, 9), less income (4), unemployment or informal sector work (10), and a higher probability of living in poverty (7).

Prenatal and postnatal growth predict childhood and adolescent cognition. A meta-analysis of 68 studies from LMICs demonstrated associations between attained linear growth in children ≤24 mo of age and cognition and motor skills at 5–11 y of age (11). In the multicountry Young Lives Study, conditional growth from 1 to 8 y of age was associated with better performance in mathematics, reading, and vocabulary tests in children aged 8 y (12). They also found that stunted children or those who were stunted at age 1 y but not at age 8 y had lower cognitive scores than their counterparts who never experienced growth faltering (12). Other analyses of the Young Lives Study data also reported that linear growth across childhood or in adolescence was associated with cognitive outcomes at ages 12 and 15 y, respectively (13, 14). In a cohort of Thai children, linear growth rates from birth to 4 mo and from 4 to 12 mo were both positively associated with intelligence quotient (IQ) at 9 y of age (15). In a cohort study of Chinese children, weight gain between 6 and 12 mo after birth showed positive associations with IQ, comprehension, memory, and reasoning at ages 7–9 y (16).

Unlike the above evidence about the role of growth in adult schooling attainment and on child IQ, fewer studies have investigated the long-term associations between growth across childhood and adult IQ. Two studies in Brazilian cohorts showed that growth in early childhood had a positive association with IQ, school attainment, and monthly income in adults (5, 17). Given the paucity of studies on this topic and the fact that LMICs have the highest burden of growth faltering in childhood, the long-term correlates of child growth on human capital need further research.

Although the importance of growth during the first 1000 d to acquisition of human capital is undisputed (4, 6–8, 18), it has been suggested that there is a second window of opportunity during adolescence when catch-up in height may occur (2, 14, 19). There is expressed need for better comprehension of the consequences of growth in this period (20). Therefore, studies are needed to assess how adult human capital is associated with growth during different age intervals from birth to adulthood, evidence that may contribute to better targeted interventions.

Our aim is to describe associations between growth across 4 age ranges [prenatal, birth to 2 y, 2 y to midchildhood (MC), and MC to adulthood] and 2 dimensions of adult human capital (school attainment and IQ) in 6 birth cohorts from 5 LMICs.

## Methods

### Study design and data sources

We analyzed data from the 6 birth cohorts that constitute the Consortium of Health-Oriented Research in Transitioning Societies (COHORTS) (21). The cohorts are from Brazil (the 1982 and 1993 Pelotas Birth Cohort Studies) (22, 23), Guatemala (the Institute of Nutrition of Central America and Panama Nutrition Trial Cohort) (24), India (the New Delhi Birth Cohort) (25), The Philippines (the Cebu Longitudinal Health and Nutrition Survey) (26), and South Africa (the Birth to Twenty Plus Cohort) (27). At the time of each cohort's establishment, India was classified as low income; Brazil (1982), Guatemala, and the Philippines were classified as lower-middle income; and Brazil (1993) and South Africa were classified as upper-middle income.

### Child growth

Anthropometrics in childhood were obtained using site-specific protocols as described elsewhere (22–27). Birth data were collected in hospitals after delivery in Pelotas 1982, Pelotas 1993 and Soweto 1990 (22, 23, 27), in the community within 3 d of birth by the research team in New Delhi between 1969 and 1972 (25), at home or in hospitals by birth attendants in Cebu between 1983 and 1984 (26), and in a healthcare centers or at home by a project nurse 15 d after birth in Guatemala between 1969 and 1977 (24). Birth weight was available in all cohorts, and birth length was available in all cohorts except the 1982 Pelotas (Brazil) and the Birth to Twenty Plus (South Africa) birth cohorts (22, 27). Calculation of gestational age was based on the date of last menstrual period reported by the child`s mother, and supplemented with Ballard scores for low–birth weight infants in the Philippines. We identified ages at measurement common across the birth cohorts as used in previous COHORTS` papers (6, 18). Supine length and weight were measured at age 24 mo in all cohorts except the 1993 Brazil birth cohort, for which measurements were obtained at 12 mo. We refer to this age as at ∼2 y or early childhood. Standing height and weight were measured at 48 mo for all cohorts (supine length was measured in Guatemala) except for the Philippines, where measures were obtained at 108 mo. We refer to this age as midchildhood (MC). We refer to all postnatal measures of length and height as height, for convenience. For descriptive purposes, heights and weights were expressed as height-for-age *z*-scores (HAZ) and weight-for-age *z*-scores (WAZ) using the WHO Growth Standards (28).

Repeated measures of height and weight were highly correlated. As in previous work (6), we created conditional height measures by regressing current height on all previous height and weight measures, and conditional relative weight measures by regressing current weight on current height and all prior height and weight measures, within strata of site and sex. Conditional size variables are standardized (mean = 0 and SD = 1) residuals of such regressions, and denote how much a child deviates from his/her expected height or weight based on his/her earlier growth, considering the growth trajectories of the other children of the same sex and cohort.

We generated 3 conditional height variables: conditional height at ∼2 y and conditional height in MC and in adulthood. These variables correspond to linear growth from birth to 2 y, from 2 y to MC, and from MC to adulthood, respectively. Regarding relative weight, 3 conditional variables were also generated: conditional weight at ∼2 y and in MC and adulthood. These variables correspond to relative weight gain from birth to 2 y, from 2 y to MC, and from MC to adulthood, respectively.

As birth length was not available for 2 cohorts, we generated conditional measures for growth (height and relative weight) using birth weight as the anchor. Additionally, results using birth length as the anchor (for the 4 cohorts for which this measure was available) are provided in the Supplementary Tables. Previous analyses have shown that models starting with birth weight or birth length produce similar associations with later outcomes (6).

### Adult outcomes

All study sites except India collected IQ data in adulthood. In Brazil in 1982, and in Guatemala, Philippines and South Africa, IQ and schooling attainment were measured when participants were 30, 47, 34, and 28 y of age, respectively. In Pelotas in 1993, IQ was measured at 18 y of age and schooling attainment was measured at 22 y of age. In India, schooling attainment was recorded at 36 y of age.

We obtained the highest grade of attained formal schooling previously collected by interview. We modeled attained schooling as an integer variable. For India, the data were only available in categorized form, and we assigned numeric values based on the years typically required to attain each category. We also categorized the number of years of schooling attainment as a binary variable, using site-specific thresholds based on the standards of each country regarding the number of years of high school completion current at the time the cohort members were children (Brazil 1982: ≥12 y; Brazil 1993: ≥11 y; Guatemala: ≥6 y; India: >12 y; The Philippines: ≥11 y; South Africa: ≥12 y).

To measure IQ in adulthood, we administered the Raven's Standard Progressive Matrices (29) to participants in the Guatemalan, Philippines, and South African cohorts. In Guatemala, sections A through C were administered due to inability to proceed beyond this point, for a maximum score of 36 points. In the Philippines and South Africa, sections A through E were administered, for a maximum score of 60 points. In both Brazilian cohorts, the arithmetic, digit symbol, similarities, and picture completion subtests of the Wechsler Adult Intelligence Scale (3rd version) were administered (30). Adult IQ was not available for the India cohort. We standardized the distribution within each cohort and by sex to a mean of 100 and an SD of 15 to remove between-cohort differences that may relate to language of administration, context, or tests administered.

### Covariates

Maternal measures of height (cm), age (years), and schooling (years) at birth of the cohort participant, paternal schooling (years), child birth order, household socioeconomic status (quintiles of the site-specific distribution), and for Guatemalan birth year and intervention group (Atole, Fresco), in the original nutrition supplementation trial near the time of the cohort participant's birth were extracted from data archives.

### Statistical analysis

We used R version 3.6.2 for analyses. We restricted all analyses to participants with complete information for anthropometric variables at birth and in childhood, and IQ (except India) and schooling variables in adulthood. For descriptive analyses, we calculated means and SDs for continuous variables, and proportions for categorical variables.

In cohort-specific analyses, we used linear regression for continuous outcomes (with theβ coefficient reflecting a change in the number of years of attained schooling or IQ standardized scores per unit change in the exposure variables, such as conditional height and conditional relative weight at each age interval) and quasi-Poisson regression for the binary outcome of school attainment. We used inverse sampling weights in all analyses of the 1993 Brazil cohort, because data were collected for all low birth weight infants and a random 20% sample of other infants.

All cohort-specific analyses were performed for male and female subjects separately. We stratified the analyses by study site and sex given observed heterogeneity among sites and previous literature that supports sex differences in IQ scores (31). Sex-combined estimates were generated by pooling the sex-specific estimates using weighted random effects meta-analysis, with the weight each sex received being proportional to sample size.

A doubly robust strategy was used for covariate adjustment, where adjustment was performed via multivariable regression after inverse probability of treatment weighting (IPTW) using the ipwpoint function from the ipw package in R (32), applying linear regression to model the relation between the exposure variable and the covariates. This regression was specified to include a “main effect” term for all covariates, as well as all pairwise product terms between covariates. To mitigate the possibility that individuals with extreme weights could substantially influence the results, the left tail of the weights was truncated at the 0.5th percentile, and the right tail at the 99.5th percentile.

We defined 4 models a priori with progressive adjustment of potential confounders. We used listwise deletion in all models. In model 1 (minimal adjustment), we adjusted for sex (for analyses involving both sexes), and year of birth and intervention group in Guatemala. In model 2 (*n* = 6961), we controlled for the same variables as in model 1 (*n* = 7989) but excluded 1028 cases that could not be included in subsequent models because of missing values. We found similar point estimates between models 1 and 2 (**Supplemental Table 1**), suggesting that missing covariate data (**Supplemental Table 2**) was not a major factor in our results. In model 3, we adjusted for the covariates in model 1, plus early-life socioeconomic quintiles, maternal schooling, maternal age at birth, maternal height, birth order and, for the Brazil 1982 and 1993 cohorts, skin color (a proxy measure of socioeconomic status and race). Comparing models 2 and 3 allowed us to assess changes due to confounding by the selected covariates. In model 4 (further adjustment), we controlled for covariates in model 3 plus paternal schooling. Model 4 is our preferred representation.

We used random effects meta-analysis to pool the sex- and cohort-specific results. Pooled sex-combined estimates were generated by pooling the corresponding pooled sex-specific estimates. The variation between cohorts was estimated using the *I*² statistic and Cochran's Q test, and random effects meta-regression was used to test for the effect modification by sex.

We defined statistical significance at an α level of 0.001 to account for the multiple comparisons carried out.

### Ethics statement

All fieldwork data collection procedures in each of the single 6-birth cohorts followed procedures approved by local Ethics Review committees. The present analyses were approved by the Emory University Institutional Review Board (IRB number 95960).

## Results

Data from 9503 participants with complete data for ≥1 of the outcomes, birth weight and at least HAZ or WAZ at ∼2 y of age were analyzed. **Table 1** shows selected characteristics of the participants. Birth weight *z*-scores were lowest among individuals born in India. The Guatemalan, Filipino, and Indian participants had the lowest height-for-age and weight-for-age *z*-scores at ∼2 y and in MC and were shortest as adults. Schooling for both parents was lowest in Guatemala. School attainment was higher in women than men across all cohorts, except in Guatemala.

**TABLE 1:** Characteristics of participants in the 6 cohorts, stratified by sex^1^

	Brazil		Brazil		Guatemala		India		Philippines		South Africa	
	1982		1993		1969–77		1969–72		1983–84		1990	
	Men	Women	Men	Women	Men	Women	Men	Women	Men	Women	Men	Women
	(*n* = 1606)	(*n* = 1657)	(*n* = 564)	(*n* = 629)	(*n* = 332)	(*n* = 394)	(*n* = 840)	(*n* = 614)	(*n* = 901)	(*n* = 813)	(*n* = 548)	(*n* = 605)
Birth variables												
Gestational age, wk	39.4 ± 1.7	39.4 ± 1.8	39.2 ± 2.6	39.1 ± 2.8	39.1 ± 2.8	39.6 ± 3.1	38.7 ± 2.6	39.1 ± 2.4	38.7 ± 2.2	38.8 ± 2.1	38.1 ± 1.8	38.0 ± 1.9
Birth length, WHO *z*-score	NA	NA	−0.8 ± 1.6	−1.0 ± 1.5	0.0 ± 1.3	0.0 ± 1.1	−0.7 ± 1.1	−0.6 ± 1.1	−0.3 ± 1.1	−0.2 ± 1.1	NA	NA
Birth weight, WHO *z*-scores	−0.2 ± 1.1	−0.2 ± 1.2	−0.7 ± 1.5	−0.9 ± 1.5	−0.7 ± 1.1	−0.6 ± 1.0	−1.1 ± 1.0	−1.1 ± 0.9	−0.7 ± 1.0	−0.6 ± 1.0	−0.6 ± 1.1	−0.6 ± 1.2
Childhood variables^2^												
HAZ at ∼2 y	−0.7 ± 1.2	−0.6 ± 1.2	−0.3 ± 1.3	−0.2 ± 1.2	−3.0 ± 1.1	−2.9 ± 1.1	−2.0 ± 1.2	−1.9 ± 1.1	−2.4 ± 1.1	−2.4 ± 1.1	−1.4 ± 1.1	−1.1 ± 1.0
HAZ in MC	−0.6 ± 1.1	−0.6 ± 1.1	−0.2 ± 1.1	−0.2 ± 1.1	−2.4 ± 0.9	−2.5 ± 1.0	−1.9 ± 1.0	−2.0 ± 1.0	−2.1 ± 0.9	−2.0 ± 0.9	−1.0 ± 0.9	−0.9 ± 0.9
WAZ at ∼2 y	0.1 ± 1.1	0.1 ± 1.0	0.4 ± 1.2	0.4 ± 1.0	−1.8 ± 1.0	−1.7 ± 1.0	−1.5 ± 1.1	−1.4 ± 1.1	−1.7 ± 1.0	−1.7 ± 1.0	−0.7 ± 1.1	−0.3 ± 1.0
WAZ in MC	0.1 ± 1.0	0.0 ± 1.0	0.3 ± 1.2	0.2 ± 1.1	−1.4 ± 0.8	−1.5 ± 0.9	−1.3 ± 0.9	−1.5 ± 0.9	−2.0 ± 1.0	−1.9 ± 0.9	−0.5 ± 0.9	−0.5 ± 0.9
Covariates												
Maternal age at childbirth, y	26.1 ± 6.2	26.2 ± 6.4	26.3 ± 6.6	26.3 ± 6.2	27.1 ± 7.2	27.3 ± 7.1	26.9 ± 6.0	26.9 ± 5.6	26.4 ± 6.1	26.4 ± 6.0	25.6 ± 6.3	25.7 ± 6.3
Maternal height, cm	156.6 ± 6.3	156.5 ± 5.8	159.7 ± 6.6	159.7 ± 6.7	148.1 ± 4.8	148.3 ± 5.1	151.8 ± 5.6	152.0 ± 5.1	150.5 ± 4.9	150.5 ± 5.0	158.2 ± 5.8	158.7 ± 5.8
Maternal schooling, y	6.5 ± 4.0	6.7 ± 4.2	6.7 ± 3.5	6.5 ± 3.4	1.3 ± 1.5	1.3 ± 1.5	5.5 ± 4.5	5.7 ± 4.5	7.0 ± 3.3	6.8 ± 3.1	9.7 ± 2.4	9.6 ± 2.6
Paternal schooling, y	6.7 ± 4.1	6.9 ± 4.2	6.6 ± 3.5	6.9 ± 4.2	1.8 ± 2.2	1.8 ± 2.1	10.4 ± 5.1	11.2 ± 4.7	7.2 ± 3.5	6.9 ± 3.3	10.6 ± 2.4	10.5 ± 2.5
Birth order, %												
1	40.6	38.9	35.6	33.1	18.5	14.0	16.4	14.0	21.8	21.8	37.4	38.7
2	28.7	29.0	24.5	28.5	12.7	14.7	23.1	20.9	21.6	23.0	29.2	28.4
3	16.0	16.0	19.1	22.1	12.1	15.0	20.7	21.4	19.6	18.9	17.0	17.4
≥4	14.7	16.1	20.7	16.4	56.7	56.3	39.8	43.8	37.0	36.3	16.4	15.5
Adult variables												
Age, y^3^	30.2 ± 0.3	30.2 ± 0.3	18.4 ± 0.3	18.4 ± 0.3	46.5 ± 2.6	46.2 ± 2.6	36.1 ± 1.1	36.1 ± 1.0	34.4 ± 0.5	34.5 ± 0.5	28.5 ± 0.4	28.5 ± 0.4
Height, cm	173.8 ± 6.9	161.1 ± 6.2	174.1 ± 7.7	160.2 ± 6.8	163.7 ± 5.8	151.1 ± 5.7	169.6 ± 6.4	154.9 ± 5.6	162.9 ± 5.9	151.1 ± 5.4	171.5 ± 6.4	159.5 ± 6.2
BMI	27.2 ± 5.1	26.8 ± 6.0	24.6 ± 4.7	25.4 ± 5.8	26.7 ± 4.3	29.4 ± 5.1	26.9 ± 4.6	27.5 ± 5.2	24.8 ± 4.4	25.2 ± 5.0	21.6 ± 3.9	25.8 ± 6.1
School attainment, y	10.9 ± 4.0	11.9 ± 4.2	9.2 ± 2.7	10.0 ± 2.3	5.6 ± 3.7	4.7 ± 3.7	13.2 ± 3.4	13.9 ± 3.1	10.2 ± 3.4	11.2 ± 2.9	11.5 ± 1.6	11.9 ± 1.4

1 Values are means ± SDs or percentages. Sample sizes (*n*) refer to participants with nonmissing data for ≥1 outcome, nonmissing data for birth weight, and at least HAZ or WAZ at 2 y of age. HAZ, height-for-age z-score; IQ, intelligence quotient; MC, midchildhood; NA, not available data; WAZ, weight-for-age z-score.

2 HAZ and WAZ measures at ∼2 y were measured at 24 mo of age in all sites except Brazil 1993, where measures were obtained at 12 mo of age. MC corresponds to 48 mo for all study sites but The Philippines, where anthropometric measures were obtained at 108 mo of age.

3 The adult age that is reported refesr to the age at which IQ was measured, except for India where it refers to the most recent age at which schooling was measured.

### Growth and school attainment

The site- and sex-pooled associations between growth and school attainment are presented in **Table 2** and **Supplemental Table 3**. Measures of weight or length at birth, and height at ∼2 y and in MC were each positively associated (*P* < 0.001) with schooling attainment. Covariate adjustment attenuated the estimates and linear growth in MC was no longer significant. For each *z*-score in birth weight and conditional height at ∼2 y of age there was an increase in 0.25 (95% CI: 0.10, 0.40) and 0.13 (95% CI: 0.08, 0.19) y of attained schooling in adulthood, respectively (Table 2). One *z*-score in birth length was also associated with an increment in the number of years of school attainment (0.17, 95% CI: 0.07, 0.27) (Supplemental Table 3). Conditional height in adulthood was not associated with schooling attainment in pooled or sex-stratified analysis. Conditional relative weights at ∼2 y in MC and in adulthood were not associated with schooling attainment in minimally and fully adjusted models. The point estimates were generally consistent between men and women; however, the association between birth weight and school attainment was the only statistically significant association (women only). The results were also consistent in direction across the 6 cohorts, although there was heterogeneity in the size of the estimates and in the statistical significance (**Table 3** and **Supplemental Table 4**). Among men in Guatemala, schooling attainment was positively associated with conditional height in adulthood and inversely associated with conditional relative weight in adulthood (Table 3). No other associations with conditional relative weight were significant. In models that examined the binary categorization of schooling attainment, we found similar associations in both pooled and site-stratified analyses (**Supplemental Tables 5,6**, and **7**).

**TABLE 2:** Pooled adjusted associations between conditional growth in childhood (birth weight as anchor) and school attainment and IQ in adulthood, by sex^1^

Models^2^	Birth weight	Conditional height at ∼2 y	Conditional height in MC	Conditional height in adulthood	Conditional relative weight at ∼2 y	Conditional relative weight in MC	Conditional relative weight in adulthood
School attainment, y							
Both sexes							
Minimally adjusted	0.25 (0.14, 0.36)*	0.70 (0.37, 1.02)*	0.25 (0.16, 0.34)*	−0.08 (−0.24, 0.09)	0.15 (0.05, 0.25)	−0.08 (−0.17, 0.00)	−0.04 (−0.53, 0.45)
Adjusted	0.13 (0.08, 0.19)*	0.25 (0.10, 0.40)*	0.09 (−0.03, 0.20)	−0.05 (−0.24, 0.14)	0.05 (−−0.03, 0.13)	−0.09 (−0.18, −0.01)	−0.13 (−0.26, −0.01)
Men							
Minimally adjusted	0.24 (0.08, 0.41)	0.76 (0.28, 1.23)	0.25 (0.15, 0.35)*	0.02 (−0.17, 0.21)	0.15 (−0.02, 0.31)	−0.08 (−0.18, 0.03)	0.22 (−0.07, 0.52)
Adjusted	0.15 (0.04, 0.27)	0.19 (−0.02, 0.40)	0.11 (−0.07, 0.28)	0.09 (−0.16, 0.33)	0.07 (−0.08, 0.22)	−0.12 (−0.24, 0.00)	−0.07 (−0.48, 0.33)
Women							
Minimally adjusted	0.26 (0.12, 0.40)*	0.64 (0.19, 1.09)	0.26 (0.02, 0.49)	−0.15 (−0.29, −0.01)	0.15 (0.03, 0.27)	−0.16 (−0.33, 0.01)	−0.28 (−0.51, −0.06)
Adjusted	0.13 (0.07, 0.19)*	0.32 (0.09, 0.54)	0.07 (−0.09, 0.23)	−0.12 (−0.21, −0.02)	0.04 (−0.06, 0.14)	0.02 (−0.19, 0.22)	−0.14 (−0.27, −0.01)
IQ score, harmonized units							
Both sexes							
Minimally adjusted	1.45 (1.14, 1.76)*	3.24 (2.35, 4.13)*	1.07 (0.69, 1.46)*	−0.37 (−0.85, 0.12)	0.30 (−0.16, 0.75)	0.08 (−0.43, 0.59)	−0.01 (−1.63, 1.60)
Adjusted	0.74 (0.35, 1.14)*	1.52 (0.96, 2.08)*	0.16 (−0.47, 0.78)	0.05 (−0.91, 1.00)	−0.26 (−1.21, 0.69)	−0.19 (−0.69, 0.32)	0.04 (−0.50, 0.58)
Men							
Minimally adjusted	1.33 (0.88, 1.78)*	3.43 (2.29, 4.58)*	0.96 (0.42, 1.51)*	−0.46 (−1.01, 0.09)	0.28 (−0.28, 0.83)	0.08 (−0.48, 0.64)	0.88 (−0.29, 2.05)
Adjusted	0.70 (0.14, 1.26)	1.66 (1.04, 2.29)*	0.00 (−0.93, 0.92)	0.00 (−1.34, 1.33)	−1.26 (−5.07, 2.55)	−0.27 (−0.82, 0.29)	0.18 (−1.57, 1.94)
Women							
Minimally adjusted	1.57 (1.13, 2.00)*	2.95 (1.54, 4.36)*	1.18 (0.65, 1.72)*	−0.04 (−1.07, 1.00)	0.35 (−0.46, 1.16)	0.06 (−1.20, 1.32)	−0.77 (−1.48, −0.07)
Adjusted	0.78 (0.23, 1.34)	1.00 (−0.19, 2.19)	0.29 (−0.56, 1.15)	0.10 (−1.28, 1.48)	−0.19 (−1.17, 0.79)	0.22 (−1.02, 1.46)	0.03 (−0.54, 0.60)

1 Values are linear regression coefficients (*β*s and 95% CIs). *P value < 0.001. IQ, intelligence quotient; MC, midchildhood.

2 In minimally adjusted models, pooled models adjusted for sex. In Guatemala analysis, we also controlled for year at birth and intervention group. In fully adjusted analyses, we controlled for maternal factors (height, age at childbirth, schooling), paternal schooling, birth order, and income/wealth quintiles. Additionally, we controlled for maternal skin color in both Brazil cohorts.

**TABLE 3:** Adjusted associations between conditional growth in childhood (birth weight as anchor) and the number of years of school attainment in adulthood, by study site and sex^1^

Sex	Birth weight	Conditional height at ∼2 y	Conditional height in MC	Conditional height in adulthood	Conditional relative weight at ∼2 y	Conditional relative weight in MC	Conditional relative weight in adulthood	*n * ^2^
Brazil 1982								
Men	0.23 (0.08, 0.39)	0.45 (0.16, 0.74)	−0.08 (−0.27, 0.10)	−0.04 (−0.26, 0.18)	0.27 (0.07, 0.46)	−0.05 (−0.22, 0.13)	−0.20 (−0.40, −0.01)	1456
Women	0.22 (0.03, 0.41)	0.51 (0.30, 0.71)*	0.07 (−0.15, 0.30)	−0.09 (−0.30, 0.12)	−0.05 (−0.25, 0.15)	−0.01 (−0.19, 0.18)	−0.30 (−0.50, −0.10)	1500
Both sexes	0.23 (0.10, 0.35)*	0.48 (0.30, 0.65)*	0.00 (−0.16, 0.15)	−0.06 (−0.22, 0.09)	0.11 (−0.19, 0.42)	−0.03 (−0.16, 0.10)	−0.25 (−0.39, −0.11)*	2956
Brazil 1993								
Men	0.18 (0.07, 0.29)	−0.07 (−0.29, 0.15)	0.34 (0.09, 0.60)	−0.18 (−0.54, 0.19)	−0.11 (−0.35, 0.12)	−0.16 (−0.45, 0.13)	0.01 (−0.25, 0.27)	403
Women	0.13 (0.05, 0.21)*	0.19 (−0.03, 0.41)	0.22 (0.01, 0.43)	−0.14 (−0.35, 0.08)	0.05 (−0.12, 0.22)	−0.01 (−0.22, 0.19)	0.03 (−0.20, 0.26)	490
Both sexes	0.15 (0.09, 0.22)*	0.07 (−0.18, 0.33)	0.28 (0.11, 0.44)*	−0.15 (−0.36, 0.05)	−0.02 (−0.18, 0.13)	−0.08 (−0.26, 0.09)	0.02 (−0.15, 0.19)	893
Guatemala								
Men	0.35 (−0.21, 0.90)	0.58 (−0.18, 1.35)	−0.50 (−1.27, 0.28)	0.92 (0.38, 1.47)*	−0.08 (−0.61, 0.46)	0.29 (−0.38, 0.96)	−1.23 (−1.70, −0.77)*	134
Women	0.27 (−0.21, 0.76)	−0.01 (−0.69, 0.67)	0.35 (−0.09, 0.78)	0.15 (−0.40, 0.71)	0.18 (−0.74, 1.11)	0.66 (−0.24, 1.56)	−0.51 (−1.15, 0.12)	148
Both sexes	0.31 (−0.06, 0.67)	0.27 (−0.30, 0.85)	−0.05 (−0.87, 0.77)	0.52 (−0.24, 1.28)	0.07 (−0.50, 0.63)	0.49 (−0.09, 1.07)	−0.83 (−1.55, −0.12)	282
India								
Men	0.53 (0.06, 0.99)	0.44 (−0.14, 1.01)	−0.13 (−0.74, 0.47)	0.10 (−0.61, 0.82)	0.56 (−0.04, 1.16)	−0.24 (−0.71, 0.24)	0.08 (−0.82, 0.99)	280
Women	−0.04 (−0.51, 0.42)	0.89 (0.30, 1.47)	0.24 (−0.20, 0.68)	−0.06 (−0.48, 0.35)	0.39 (0.01, 0.77)	−0.37 (−0.74, 0.00)	−0.25 (−0.54, 0.04)	243
Both sexes	0.26 (−0.30, 0.82)	0.65 (0.20, 1.09)	0.04 (−0.34, 0.42)	0.03 (−0.40, 0.45)	0.48 (0.12, 0.85)	−0.30 (−0.60, 0.01)	−0.07 (−0.58, 0.43)	523
Philippines								
Men	0.09 (−0.14, 0.31)	0.29 (0.00, 0.57)	0.23 (0.00, 0.46)	−0.17 (−0.42, 0.09)	−0.06 (−0.31, 0.18)	−0.20 (−0.50, 0.10)	0.41 (0.11, 0.70)	877
Women	0.11 (−0.16, 0.38)	0.44 (0.21, 0.67)*	−0.01 (−0.19, 0.18)	0.01 (−0.20, 0.22)	0.12 (−0.16, 0.41)	−0.21 (−0.47, 0.05)	−0.09 (−0.32, 0.14)	783
Both sexes	0.10 (−0.08, 0.27)	0.36 (0.17, 0.54)*	0.12 (−0.12, 0.36)	−0.08 (−0.26, 0.09)	0.02 (−0.16, 0.21)	−0.20 (−0.40, 0.00)	0.18 (−0.31, 0.67)	1660
South Africa								
Men	0.00 (−0.12, 0.11)	−0.02 (−0.15, 0.11)	0.18 (−0.01, 0.36)	0.22 (−0.01, 0.44)	0.05 (−0.09, 0.20)	−0.32 (−0.66, 0.03)	0.47 (0.08, 0.87)	309
Women	0.10 (−0.02, 0.21)	0.02 (−0.15, 0.19)	−0.16 (−0.31, −0.02)*	−0.21 (−0.37, −0.06)*	−0.04 (−0.28, 0.20)	0.01 (−0.14, 0.17)	−0.02 (−0.23, 0.18)	338
Both sexes	0.05 (−0.05, 0.15)	0.00 (−0.10, 0.11)	0.00 (−0.33, 0.33)	−0.01 (−−0.43, 0.41)	0.00 (−0.14, 0.15)	−0.15 (−0.47, 0.17)	0.21 (−0.27, 0.70)	647
All sites^3^								
Men	0.15 (0.04, 0.27)	0.19 (−0.02, 0.40)	0.11 (−0.07, 0.28)	0.09 (−0.16, 0.33)	0.07 (−0.08, 0.22)	−0.12 (−0.24, 0.00)	−0.07 (−0.48, 0.33)	3459
*I* ² statistic, %	54.5	67.7	57.5	70.7	51.6	0	88.4	
Cochran's Q test, *P* value	0.051	0.008	0.038	0.004	0.067	0.566	<0.001	
All sites								
Women	0.13 (0.07, 0.19)*	0.32 (0.09, 0.54)	0.07 (−0.09, 0.23)	−0.12 (−0.21, −0.02)	0.04 (−−0.06, 0.14)	−0.06 (−0.18, 0.07)	−0.14 (−0.27, −0.01)	3502
*I* ² statistic, %	0	75.7	62.2	0	0	34.2	34.9	
Cochran's Q test, *P* value	0.83	0.001	0.021	0.55	0.436	0.179	0.175	
All sites								
Both sexes	0.13 (0.08, 0.19)*	0.25 (0.10, 0.40)*	0.09 (−0.03, 0.20)	−0.05 (−0.24, 0.14)	0.05 (−0.03, 0.13)	−0.09 (−0.18, −0.01)	−0.13 (−0.26, −0.01)	6961
*I* ² statistic, %	16.3	74.4	59.6	56.2	28.2	12.9	78.8	
Cochran's Q test, *P* value	0.284	<0.001	0.004	0.009	0.169	0.319	<0.001	

1 Values are linear regression coefficients (*β*s and 95% CIs). In adjusted analyses, we controlled for maternal factors (height, age at childbirth, schooling), birth order, and income/wealth quintiles. Additionally, we controlled for year at birth and intervention group in Guatemala, and for maternal skin color in both Brazil cohorts. *P value < 0.001. MC, midchildhood.

2 These models exclude cases with missing values.

3 These β coefficients are the same as presented in Table 2 (adjusted models). The *I* ² statistic and Cochran's Q test were used to quantify between-cohort variation.

### Growth and IQ

The site- and sex-pooled associations between growth and IQ are presented in Table 2 and Supplemental Table 3. Measures of weight or length at birth, and height at ∼2 y and in MC were each positively associated (*P* < 0.001) with adult IQ. After covariate adjustment the strength of associations was reduced and the estimate for conditional height in MC was not significant. For each *z*-score in birth weight and conditional height at ∼2 y of age we observed 0.74 (95% CI: 0.35, 1.14) and 1.52 (95% CI: 0.96, 2.08) more units in adult IQ score, respectively (Table 2). One *z*-score in birth length was associated with 0.73 (95% CI: 0.35, 1.10) more units in adult IQ score (Supplemental Table 3). Conditional height in adulthood was not associated with IQ. Measures of relative weight at ∼2 y, in MC, and in adulthood were not associated with adult IQ. In general, the results were consistent between males and females in minimally adjusted models. However, in fully adjusted models only the association between conditional height at ∼2 y was statistically significant (men). There was heterogeneity across the 5 cohorts in the size and significance of the estimates (**Table 4** and **Supplemental Table 8**).

**TABLE 4:** Adjusted associations between conditional growth in childhood (birth weight as anchor) and IQ in adulthood, by study site and sex^1^

Sex	Birth weight	Conditional height at ∼2 y	Conditional height in MC	Conditional height in adulthood	Conditional relative weight at ∼2 y	Conditional relative weight in MC	Conditional relative weight in adulthood	*n* ^2^
Brazil 1982								
Men	0.36 (−0.31, 1.03)	1.94 (1.10, 2.79)*	−0.15 (−0.89, 0.59)	0.32 (−0.80, 1.43)	0.58 (−0.17, 1.32)	−0.05 (−0.77, 0.67)	0.48 (−0.21, 1.18)	1445
Women	1.26 (0.59, 1.92)*	1.13 (0.32, 1.93)	0.38 (−0.39, 1.15)	−0.09 (−0.83, 0.64)	−0.11 (−0.84, 0.62)	−0.26 (−1.04, 0.52)	−0.05 (−0.85, 0.75)	1492
Both sexes	0.81 (−0.07, 1.69)	1.53 (0.73, 2.33)*	0.12 (−0.41, 0.65)	0.11 (−0.56, 0.77)	0.24 (−0.44, 0.91)	−0.15 (−0.68, 0.38)	0.22 (−0.31, 0.75)	2937
Brazil 1993								
Men	1.06 (0.42, 1.70)*	2.52 (0.70, 4.34)	1.62 (−0.25, 3.50)	0.46 (−1.05, 1.98)	0.40 (−1.90, 2.69)	−0.65 (−2.25, 0.96)	−0.80 (−2.71, 1.11)	381
Women	0.51 (−0.06, 1.09)	0.42 (−0.93, 1.77)	1.36 (−0.09, 2.81)	0.12 (−1.40, 1.64)	0.88 (−0.60, 2.36)	0.14 (−1.49, 1.78)	0.21 (−1.33, 1.76)	466
Both sexes	0.78 (0.25, 1.31)	1.37 (−0.68, 3.41)	1.48 (0.32, 2.64)	0.27 (−0.81, 1.35)	0.66 (−0.65, 1.98)	−0.21(−1.36, 0.94)	−0.24 (−1.45, 0.97)	847
Guatemala								
Men	2.04 (−0.19, 4.26)	2.21 (−0.44, 4.86)	−3.02 (−7.23, 1.18)	3.70 (0.38, 7.03)	−7.22 (−8.00, −6.44)*	−1.60 (−5.26, 2.06)	−2.33 (−3.08, −1.58)*	130
Women	−0.74 (−2.54, 1.07)	−2.36 (−5.67, 0.94)	1.09 (−1.78, 3.96)	1.76 (−1.87, 5.39)	−2.66 (−5.59, 0.28)	1.17 (−2.52, 4.87)	0.23 (−1.87, 2.33)	147
Both sexes	0.54 (−2.17, 3.25)	−0.22 (−4.72, 4.29)	−0.84 (−4.84, 3.16)	2.67 (0.19, 5.15)	−4.66 (−9.22, −0.11)	−0.05 (−2.79, 2.69)	−0.90 (−3.47, 1.68)	277
India								
Men	NA	NA	NA	NA	NA	NA	NA	NA
Women	NA	NA	NA	NA	NA	NA	NA	NA
Both sexes	NA	NA	NA	NA	NA	NA	NA	NA
Philippines								
Men	−0.21 (−1.37, 0.95)	0.55 (−0.78, 1.88)	0.38 (−0.84, 1.60)	−1.90 (−3.10, −0.71)	−0.29 (−1.48, 0.90)	0.32 (−0.95, 1.58)	1.31 (−0.27, 2.90)	672
Women	1.16 (−0.18, 2.50)	2.83 (1.61, 4.05)*	−1.18 (−2.49, 0.14)	2.00 (0.44, 3.56)	0.70 (−0.70, 2.10)	−1.42 (−2.81, −0.03)	0.00 (−1.46, 1.45)	576
Both sexes	0.42 (−0.92, 1.76)	1.60 (−0.64, 3.84)	−0.34 (−1.87, 1.19)	−0.10 (−3.93, 3.73)	0.16 (−0.80, 1.12)	−0.47 (−2.18, 1.23)	0.71 (−0.58, 2.01)	1248
South Africa								
Men	1.14 (−0.08, 2.36)	1.43 (−0.31, 3.18)	−1.37 (−3.55, 0.81)	−0.56 (−2.24, 1.13)	0.41 (−1.24, 2.06)	−1.41 (−2.92, 0.09)	2.54 (0.87, 4.22)	309
Women	1.07 (−0.97, 3.12)	0.76 (−1.47, 2.98)	0.44 (−1.27, 2.14)	−3.00 (−5.20, −0.79)*	−1.83 (−3.89, 0.24)	2.60 (0.77, 4.42)	0.06 (−1.46, 1.57)	339
Both sexes	1.11 (−0.12, 2.33)	1.08 (−0.35, 2.51)	−0.43 (−2.19, 1.33)	−1.83 (−4.24, 0.57)	−0.76 (−2.96, 1.44)	0.69 (−3.25, 4.62)	1.24 (−1.19, 3.68)	648
All sites^3^								
Men	0.70 (0.14, 1.26)	1.66 (1.04, 2.29)*	0.00 (−0.93, 0.92)	0.00 (−1.34, 1.33)	−1.26 (−5.07, 2.55)	−0.27 (−0.82, 0.29)	0.18 (−1.57, 1.94)	2937
*I* ² statistic, %	38	4.2	42.3	72.8	98.3	2.8	91.7	
Cochran's Q test, *P* value	0.168	0.383	0.14	0.005	<0.001	0.391	<0.001	
All sites								
Women	0.78 (0.23, 1.34)	1.00 (−0.19, 2.19)	0.29 (−0.56, 1.15)	0.10 (−1.28, 1.48)	−0.19 (−1.17, 0.79)	0.22 (−1.02, 1.46)	0.03 (−0.54, 0.60)	3020
*I* ² statistic, %	32	68.6	45	72.2	53	68.1	0	
Cochran's Q test, *P* value	0.208	0.013	0.122	0.006	0.075	0.014	0.998	
All sites								
Both sexes	0.74 (0.35, 1.14)*	1.52 (0.96, 2.08)*	0.16 (−0.47, 0.78)	0.05 (−0.91, 1.00)	−0.26 (−1.21, 0.69)	−0.19 (−0.69, 0.32)	0.04 (−0.50, 0.58)	5957
*I* ² statistic, %	27.7	49.8	38.8	69.9	96.8	46.6	81.9	
Cochran's Q test, *P* value	0.19	0.036	0.1	<0.001	<0.001	0.051	<0.001	

1 Values are adjusted linear regression coefficients (*β*s and 95% CIs). In adjusted analyses, we controlled for maternal factors (height, age at childbirth, schooling), birth order, and income/wealth quintiles. Additionally, we controlled for year at birth and intervention group in Guatemala, and for maternal skin color in both Brazil cohorts. *P value < 0.001. IQ, Intelligence quotient; MC, midchildhood; NA, not available data (IQ not available in India).

2 These models exclude cases with missing values.

3 These β coefficients are the same as presented in Table 2 (adjusted models). The *I* ² statistic and Cochran's Q test were used to quantify between-cohort variation.

## Discussion

This analysis of data from 6 birth cohorts from LMICs showed that birth size and linear growth from birth to age 2 y were positively associated with schooling attainment and adult IQ. Linear growth from age 2 y to MC was not associated with either of the outcomes in fully adjusted models. Conditional height in adulthood (which reflects further growth from MC through to attained adult height) was not associated with either schooling attainment or IQ. Conditional relative weights in MC and in adulthood were not associated with schooling attainment. Change in relative weight was not associated with adult IQ for any of the age intervals examined.

Our results suggest that birth size and linear growth from birth to ∼2 y are independent predictors of schooling and intelligence in adulthood because, by design, they are uncorrelated with each other. This independent association, at least in childhood, has previously been observed (12, 13, 33, 34). We found that for each *z*-score increase in birth weight, birth length, and linear growth at ∼2 y of age there was an increase in 0.13, 0.17, and 0.25 y of schooling, respectively. After adjustment, we observed that for each *z*-score increase in birth size (weight and length) and conditional height at ∼2 y of age there was an increase in 0.74, 0.73, and 1.52 units of adult IQ score, respectively (Table 2 and Supplemental Table 3). This is important because improvements in human capital dimensions are associated with economic growth. It has been estimated that for every additional year of schooling there is a 7.9% country level economic return (9); and a 1-SD increase in cognitive skills of a country`s workforce has been associated with an increase of 2 percentage points in the per capita GDP (annual growth) and economic returns that range from 0.07 to 0.48, in developing countries (35, 36).

Our findings further confirm that timing (specifically the first 1000 d) is critical to improve schooling outcomes and intellectual performance in adulthood. Moreover, our results are consistent with previous analysis that examined stunting in early childhood and adult human capital (schooling attainment, risk of failing at least on grade, age at school enrollment, performance in verbal and nonverbal cognitive tests, household expenditure, and probability of living under poverty conditions) (7, 18). Human growth is not uniform and systems, organs, and tissues develop at different velocities. Linear growth is characterized by high initial velocity with rapid deceleration in the first 2 y after birth (37). The brain achieves 83% of its adult volume by 24 mo of postnatal life (38). Thus, shared underlying determinants of both length and neurodevelopment might influence adult human capital defined as a group of capacities, abilities, and intangible assets useful to create economic value (39). Interventions in early life have the highest overall returns compared with other life course stages, and interventions to reduce stunting in the first 24 mo of age not only increased preschool linear growth but had benefit-cost ratios >1, demonstrating that such interventions are a good economic investment (20). In that sense, that benefit in linear growth also indirectly benefits schooling attainment and cognitive performance in the long term.

We infer that the multiple causes of linear growth partly contribute to brain development and learning; hence, linear growth is an indirect predictor of schooling and IQ. Thes relation between growth and adult human capital have at least 4 underlying pathways that explain the results observed. First, linear growth and brain development are susceptible to common nutritional inputs (40), which in turn are determined by constructs like socioeconomic status (poverty), maternal education, food insecurity, water scarcity, poor sanitation, and hygiene, among others (41). Thus, nutritional deficits in sensitive periods of life affect not only body growth but brain size, structure, development, and function (42). Second, growth faltering makes children more susceptible to infections that in turn decrease appetite, absorption, and nutrient use; nutrients will be diverted to the immune system, affecting their availability for growth and development (43). Third, undernourished and ill children are commonly apathetic, irritable, and less interested in exploring their environment (43). Fourth, the size of children may elicit different interactions with adults given that short children appear younger and are treated as such (44). Thus, the last 2 pathways explain why short children lag in acquiring motor, cognitive, and social behaviors (44).

Our results confirm earlier research that prenatal growth and linear growth in early childhood are significantly associated with higher school attainment (4–7, 17, 18) and adult IQ (5, 7, 17) whilst linear growth from 2 y to MC and from MC to adulthood (the period that includes late childhood, adolescence, and, adulthood through ages 18 to 46 y) are not significantly associated with schooling attainment and adult IQ (5, 6, 17). Thus, growth during adolescence as a whole is shown not to be associated with the outcomes. Analyses of Young Lives Study data have shown that linear growth in different age intervals (1, 5, 8, 12, and 15 y) was positively associated with cognitive skills in MC, early adolescence, and adolescence (12–14, 33). Despite that finding, we observed similar tendencies in the direction of the associations (12, 14, 33), and most of our estimates were not statistically significant after 2 y of age. We found that conditional height in adulthood was positively associated with schooling, while change in relative weight from MC to adulthood was inversely associated with schooling attainment only in male participants from Guatemala. We infer that linear growth and neurodevelopment are largely complete by adulthood when individuals have already finished school. Thus, it is unlikely that weight changes in adulthood are predictors of schooling attainment. Rather, the inverse associations observed in Guatemala may reflect reverse causality. In adulthood, men`s weight and weight changes are influenced by multiple biological and environmental factors, such as genetics (body composition), occupation, marital status, lifestyle behaviors, work settings, food environment, and socioeconomic status (45), many of which might be determined by schooling attainment earlier in life.

We did not find statistical associations between conditional relative weight at ∼2 y and school attainment after full adjustment. Adair et al. (6) had previously observed a significant but weak association between conditional relative weight in early childhood and the highest grade attained. The differences in our study might be attributed to the inclusion of a new birth cohort, Brazil 1993 (23), variations in sample sizes, and a larger number of covariates. Conditional relative weight at ∼2 y and in MC were not associated with IQ, as previously shown in studies conducted with adults (5, 17) and children (15).

This study has several strengths. We analyzed 6 well-characterized population-based birth cohorts in 5 LMICs with follow-up periods that ranged from 18 to 46 y. Each study site had trained staff who followed standard methodologies to collect the anthropometric and sociodemographic data, which minimized measurement error and recall bias. The treatment of our exposures as conditional measures of growth avoids collinearity and facilitates differentiation of linear growth at specific age intervals. Each site used validated measures of intelligence (29, 46), and we standardized the distributions within each cohort and by sex to be able to compare outcomes. We adjusted our models for a range of early life social factors. Additionally, our analytic sample with complete data was not affected by missing values and loss at follow-up, as evidenced by the similar results obtained when comparing models 1 and 2 (Supplemental Table 1). We saw evidence of heterogeneity among the cohorts in the magnitude of the estimates but not in the direction of the associations. Thus, we were able to obtain single pooled estimates combining cohort-specific results through meta-analysis.

Among the study's limitations, we noted some inconsistencies in the ages of exposures and outcomes across the cohorts. Height was measured at 2 y of age at all sites except the Brazil 1993 cohort, where it was measured at 1 year of age. Similarly, MC was considered to be 4 y in most of the cohorts, but 8.5 y in the Philippines. There were also differences in the ages in which the schooling and IQ outcomes were obtained, and some differences in the instruments used to measure IQ across sites. In the specific case of Brazil 1993, schooling attainment was measured at 22 y, an age at which some participants might have not finished school yet, as mentioned by Menezes et al. (17). IQ and adult height were measured when the Brazil 1993 cohort participants were 18 y old; an age at which there is a possibility that individuals might not have reached their maximum linear growth. Residual and unmeasured confounding should be considered given the observational nature of our study. The cohorts do not have information for acute or chronic inflammation in childhood because these were not recognized as important conditions when the studies were launched. We recognize that aspects of the home environment, such as psychosocial stimulation, which have been shown to influence adult human capital (47), might have some underlying role. Finally, we acknowledge that our findings might not represent the entire diversity and complexity of the studied countries, limiting their generalizability to other LMICs. These findings should also be interpreted with caution given the differences between pooled and site-specific results.

In conclusion, our results show an independent association between prenatal growth and linear growth from birth until the second year of life with adult human capital. These findings confirm the importance of the first 1000 d, a sensitive period where adversities including poor nutrition will have long-lasting effects on adult size and functional capacities such as learning and intelligence. Our findings did not show that linear growth after the first 2 y (MC to adulthood) were associated with adult human capital outcomes.

## Supplementary Material

nxab096_Supplemental_File

## Data Availability

Data will be available upon reasonable request to the principal investigators at each study site.

## Notes

Funded by the Bill and Melinda Gates Foundation (OPP1164115) for data collection in Guatemala, Philippines, and South Africa and India for data management and analysis. Data collection in Brazil was funded by the Wellcome Trust (086974/Z/08/Z). The New Delhi Birth Cohort has also received funding from the Indian Council of Medical Research (50/1-3/TF/05-NCD-II; 3/1/2/2/15-RCH; 5/10/FR/10/2019-RCH; 5/4/8-7/2019-NCD-II), the Department of Biotechnology (BT/PR3874/MED/97/1/2011; BT/PR5317/FNS/20/552/2012), the United States National Center for Health Statistics (PL-480, RESEARCH PROJECT 0-1-658-2), and the British Heart Foundation (UKPG/05/046). The Birth to Twenty Plus Cohort is supported by the South African Medical Research Council, DSI-NRF Centre of Excellence in Human Development at the University of the Witwatersrand, Johannesburg, South Africa, and the Wellcome Trust (077210/Z/05/Z; 092097/Z/10/Z). NEP is in part supported by a Fulbright-Colciencias fellowship.

Author disclosure: The authors report no conflicts of interest. ADS is a member of the Journal's Editorial Board and played no role in the Journal's evaluation of the manuscript.

Supplemental Tables 1–8 are available from the “Supplementary Data” link in the online posting of the article and from the same link in the online table of contents at https://academic.oup.com/jn.

Abbreviations used: HAZ, height-for-age *z*-score; IQ, Intelligence Quotient; LMIC, low- and middle-income country; MC, midchildhood; WAZ, weight-for-age *z*-score.
