# Supplementary material for: Patterns of Growth in Childhood in Relation to Adult Schooling Attainment and Intelligence Quotient in 6 Birth Cohorts in Low- and Middle-Income Countries: Evidence from the Consortium of Health-Oriented Research in Transitioning Societies (COHORTS)
Source: J Nutr. 2021 May 12;151(8):2342–52. doi: 10.1093/jn/nxab096 (PMC8436131; doi:10.1093/jn/nxab096)
Supplement: nxab096_Supplemental_File [file nxab096_Supplemental_File.docx]

**Patterns of growth in childhood in relation to adult schooling attainment and IQ in 6 birth cohorts in low and middle-income countries: evidence from COHORTS**

**Natalia E Poveda et al. Online Supplementary Material**

**Supplemental Table 1. Minimally adjusted associations between conditional growth in childhood (birth weight as anchor) and school attainment and IQ in adulthood, by sex^1^**

|  | **Sex** | **Models^2^** | ***n*** | **Birth weight** | **Conditional height at around 2 years of age** | **Conditional height in mid-childhood** | **Conditional height in adulthood** | **Conditional relative weight at around 2 years of age** | **Conditional relative weight in mid-childhood** | **Conditional relative weight in adulthood** |
| --- | --- | --- | --- | --- | --- | --- | --- | --- | --- | --- |
| **School attainment (years of schooling)** | Both sexes | Model 1 | 7989 | 0.26 (0.16, 0.35)* | 0.72 (0.40, 1.04)* | 0.22 (0.14, 0.31)* | -0.08 (-0.23, 0.06) | 0.17 (0.05, 0.28) | -0.06 (-0.12, 0.01) | -0.04 (-0.53, 0.45) |
|  |  | Model 2 | 6961 | 0.25 (0.14, 0.36)* | 0.70 (0.37, 1.02)* | 0.25 (0.16, 0.34)* | -0.08 (-0.24, 0.09) | 0.15 (0.05, 0.25) | -0.08 (-0.17, 0.00) | -0.04 (-0.53, 0.45) |
|  | Men | Model 1 | 4071 | 0.24 (0.09, 0.38)* | 0.79 (0.35, 1.24)* | 0.21 (0.13, 0.30)* | 0.00 (-0.16, 0.17) | 0.17 (0.00, 0.33) | -0.05 (-0.14, 0.05) | 0.22 (-0.04, 0.48) |
|  |  | Model 2 | 3459 | 0.24 (0.08, 0.41) | 0.76 (0.28, 1.23) | 0.25 (0.15, 0.35)* | 0.02 (-0.17, 0.21) | 0.15 (-0.02, 0.31) | -0.08 (-0.18, 0.03) | 0.22 (-0.07, 0.52) |
|  | Women | Model 1 | 3918 | 0.28 (0.15, 0.40)* | 0.64 (0.17, 1.11) | 0.29 (0.07, 0.51) | -0.15 (-0.27, -0.03) | 0.17 (0.01, 0.32) | -0.07 (-0.16, 0.03) | -0.28 (-0.50, -0.07) |
|  |  | Model 2 | 3502 | 0.26 (0.12, 0.40)* | 0.64 (0.19, 1.09) | 0.26 (0.02, 0.49) | -0.15 (-0.29, -0.01) | 0.15 (0.03, 0.27) | -0.16 (-0.33, 0.01) | -0.28 (-0.51, -0.06) |
| **School attainment (binary variable) ^3^** | Both sexes | Model 1 | 7989 | 1.07 (1.05, 1.09)* | 1.18 (1.12, 1.23)* | 1.06 (1.03, 1.08)* | 0.98 (0.95, 1.02) | 1.04 (1.00, 1.08) | 1.00 (0.98, 1.01) | 0.99 (0.89, 1.10) |
|  |  | Model 2 | 6961 | 1.07 (1.04, 1.09)* | 1.18 (1.12, 1.24)* | 1.06 (1.03, 1.09)* | 0.99 (0.94, 1.04) | 1.03 (0.99, 1.08) | 1.00 (0.98, 1.02) | 0.98 (0.88, 1.10) |
|  | Men | Model 1 | 4071 | 1.06 (1.03, 1.10)* | 1.20 (1.12, 1.28)* | 1.06 (1.03, 1.08)* | 1.00 (0.96, 1.05) | 1.06 (1.02, 1.10) | 0.99 (0.96, 1.01) | 1.04 (0.98, 1.11) |
|  |  | Model 2 | 3459 | 1.07 (1.03, 1.11)* | 1.20 (1.11, 1.29)* | 1.06 (1.02, 1.10) | 1.02 (0.96, 1.07) | 1.06 (1.01, 1.11) | 0.99 (0.95, 1.03) | 1.04 (0.98, 1.11) |
|  | Women | Model 1 | 3918 | 1.07 (1.05, 1.10)* | 1.16 (1.08, 1.24)* | 1.06 (1.02, 1.11) | 0.97 (0.94, 1.00) | 1.02 (0.99, 1.05) | 1.00 (0.98, 1.03) | 0.94 (0.88, 1.00) |
|  |  | Model 2 | 3502 | 1.07 (1.03, 1.10)* | 1.17 (1.09, 1.25)* | 1.06 (1.01, 1.12) | 0.96 (0.93, 1.00) | 1.01 (0.98, 1.04) | 1.01 (0.98, 1.04) | 0.93 (0.87, 0.99) |
| **IQ score (harmonized units)** | Both sexes | Model 1 | 6180 | 1.52 (1.19, 1.84)* | 3.19 (2.24, 4.13)* | 1.02 (0.65, 1.39)* | -0.28 (-0.76, 0.20) | 0.26 (-0.21, 0.73) | 0.2 (-0.34, 0.74) | 0.11 (-1.40, 1.61) |
|  |  | Model 2 | 5957 | 1.45 (1.14, 1.76)* | 3.24 (2.35, 4.13)* | 1.07 (0.69, 1.46)* | -0.37 (-0.85, 0.12) | 0.30 (-0.16, 0.75) | 0.08 (-0.43, 0.59) | -0.01 (-1.63, 1.60) |
|  | Men | Model 1 | 3053 | 1.31 (0.84, 1.78)* | 3.39 (2.16, 4.63)* | 0.86 (0.33, 1.39)* | -0.33 (-0.88, 0.21) | 0.28 (-0.26, 0.83) | 0.24 (-0.37, 0.84) | 0.94 (-0.23, 2.10) |
|  |  | Model 2 | 2937 | 1.33 (0.88, 1.78)* | 3.43 (2.29, 4.58)* | 0.96 (0.42, 1.51)* | -0.46 (-1.01, 0.09) | 0.28 (-0.28, 0.83) | 0.08 (-0.48, 0.64) | 0.88 (-0.29, 2.05) |
|  | Women | Model 1 | 3127 | 1.65 (1.29, 2.00)* | 2.89 (1.42, 4.36)* | 1.18 (0.65, 1.70)* | -0.11 (-1.11, 0.89) | 0.21 (-0.71, 1.12) | 0.08 (-1.11, 1.27) | -0.60 (-1.39, 0.19) |
|  |  | Model 2 | 3020 | 1.57 (1.13, 2.00)* | 2.95 (1.54, 4.36)* | 1.18 (0.65, 1.72)* | -0.04 (-1.07, 1.00) | 0.35 (-0.46, 1.16) | 0.06 (-1.20, 1.32) | -0.77 (-1.48, -0.07) |

^1^ Values are linear regression coefficients (*β*s and 95% CIs) for continuous variables and risk ratios (95% CIs) for categorical variables. IQ, intelligence quotient. **P* value < 0.001.

^2^ In minimally adjusted model 1, pooled models adjust for sex. In Guatemala analysis, we also controlled for year at birth and intervention group. In minimally adjusted model 2, we controlled for the same variables as in model 1 but excluded cases that could not be included in subsequent models because of missing values.

^3^ We used site-specific thresholds relevant to high school completion current at the time the cohort members were children (Brazil 1982: ≥ 12 years; Brazil 1993: ≥ 11 years; Guatemala: ≥ 6 years; India: > 12 years; The Philippines: ≥ 11 years; South Africa: ≥ 12 years).

**Supplemental Table 2. Proportion (%) of missing covariate values per cohort**

| **Denominator** | **Cohort** | **Sex** | **Income/wealth quintiles** | **Maternal age** | **Maternal height** | **Maternal schooling** | **Atole/Fresco^1^** | **Birth year^1^** | **Weights^2^** | **Skin color^3^** | **Birth order** |
| --- | --- | --- | --- | --- | --- | --- | --- | --- | --- | --- | --- |
| Entire sample | Brazil 1982 | 0.0 | 0.0 | 0.0 | 1.8 | 0.1 | 100.0 | 100.0 | 100.0 | 0.0 | 0.0 |
| Entire sample | Brazil 1993 | 0.0 | 2.1 | 0.0 | 0.9 | 0.1 | 100.0 | 100.0 | 0.0 | 0.0 | 0.0 |
| Entire sample | Guatemala | 0.0 | 13.3 | 2.0 | 24.8 | 9.3 | 0.0 | 0.0 | 100.0 | 100.0 | 2.2 |
| Entire sample | India | 0.0 | 27.5 | 28.4 | 74.4 | 23.4 | 100.0 | 100.0 | 100.0 | 100.0 | 37.5 |
| Entire sample | Philippines | 0.0 | 0.7 | 0.0 | 0.0 | 0.0 | 100.0 | 100.0 | 100.0 | 100.0 | 0.0 |
| Entire sample | South Africa | 0.0 | 12.6 | 0.1 | 46.3 | 10.4 | 100.0 | 100.0 | 100.0 | 100.0 | 0.0 |
| Those with non-missing values for both length at 2 y and schooling | Brazil 1982 | 0.0 | 0.0 | 0.0 | 1.4 | 0.1 | 100.0 | 100.0 | 100.0 | 0.0 | 0.0 |
| Those with non-missing values for both length at 2 y and schooling | Brazil 1993 | 0.0 | 1.1 | 0.0 | 1.1 | 0.2 | 100.0 | 100.0 | 0.0 | 0.0 | 0.0 |
| Those with non-missing values for both length at 2 y and schooling | Guatemala | 0.0 | 2.5 | 0.3 | 5.4 | 1.8 | 0.0 | 0.0 | 100.0 | 100.0 | 0.3 |
| Those with non-missing values for both length at 2 y and schooling | India | 0.0 | 30.7 | 32.0 | 37.0 | 10.1 | 100.0 | 100.0 | 100.0 | 100.0 | 37.1 |
| Those with non-missing values for both length at 2 y and schooling | Philippines | 0.0 | 0.4 | 0.0 | 0.0 | 0.0 | 100.0 | 100.0 | 100.0 | 100.0 | 0.0 |
| Those with non-missing values for both length at 2 y and schooling | South Africa | 0.0 | 3.7 | 0.0 | 15.4 | 3.9 | 100.0 | 100.0 | 100.0 | 100.0 | 0.0 |
| Those with non-missing values for both conditional length at 2 y and schooling | Brazil 1982 | 0.0 | 0.0 | 0.0 | 1.5 | 0.1 | 100.0 | 100.0 | 100.0 | 0.0 | 0.0 |
| Those with non-missing values for both conditional length at 2 y and schooling | Brazil 1993 | 0.0 | 0.7 | 0.0 | 0.8 | 0.2 | 100.0 | 100.0 | 0.0 | 0.0 | 0.0 |
| Those with non-missing values for both conditional length at 2 y and schooling | Guatemala | 0.0 | 0.9 | 0.0 | 0.0 | 0.4 | 0.0 | 0.0 | 100.0 | 100.0 | 0.0 |
| Those with non-missing values for both conditional length at 2 y and schooling | India | 0.0 | 31.0 | 32.2 | 36.9 | 9.9 | 100.0 | 100.0 | 100.0 | 100.0 | 36.9 |
| Those with non-missing values for both conditional length at 2 y and schooling | Philippines | 0.0 | 0.4 | 0.0 | 0.0 | 0.0 | 100.0 | 100.0 | 100.0 | 100.0 | 0.0 |
| Those with non-missing values for both conditional length at 2 y and schooling | South Africa | 0.0 | 4.0 | 0.0 | 15.4 | 4.1 | 100.0 | 100.0 | 100.0 | 100.0 | 0.0 |
| ^1^ Only applicable to Guatemala |  |  |  |  |  |  |  |  |  |  |  |
| ^2^ Only applicable to Brazil 1993. |  |  |  |  |  |  |  |  |  |  |  |
| ^3^ Only applicable to Brazil 1982 and 1993. |  |  |  |  |  |  |  |  |  |  |  |

**Supplemental Table 3. Pooled adjusted associations between conditional growth in childhood (birth length as anchor) and school attainment and IQ in adulthood, by sex^1^**

|  | **Sex** | **Models^2^** | **Birth length** | **Conditional birth weight** | **Conditional height at around 2 years of age** | **Conditional height in mid-childhood** | **Conditional height in adulthood** | **Conditional relative weight at around 2 years of age** | **Conditional relative weight in mid-childhood** | **Conditional relative weight in adulthood** |
| --- | --- | --- | --- | --- | --- | --- | --- | --- | --- | --- |
| **School attainment (years of schooling)** | Both sexes | Minimally adjusted | 0.21 (0.15, 0.27)* | 0.09 (-0.10, 0.27) | 0.70 (0.36, 1.03)* | 0.24 (0.15, 0.33)* | -0.10 (-0.26, 0.05) | 0.14 (0.04, 0.24) | -0.08 (-0.17, 0.01) | -0.03 (-0.55, 0.48) |
|  |  | Adjusted | 0.17 (0.07, 0.27)* | 0.04 (-0.18, 0.26) | 0.35 (0.17, 0.53)* | 0.12 (-0.04, 0.28) | -0.06 (-0.19, 0.07) | 0.07 (-0.03, 0.16) | -0.09 (-0.18, -0.01) | -0.02 (-0.38, 0.33) |
|  | Men | Minimally adjusted | 0.25 (0.12, 0.39)* | 0.18 (-0.18, 0.54) | 0.76 (0.28, 1.23) | 0.25 (0.15, 0.35)* | -0.01 (-0.18, 0.17) | 0.14 (-0.03, 0.30) | -0.08 (-0.19, 0.03) | 0.24 (-0.06, 0.54) |
|  |  | Adjusted | 0.19 (0.07, 0.32) | 0.18 (-0.18, 0.54) | 0.28 (0.00, 0.56) | 0.22 (0.00, 0.45) | 0.03 (-0.14, 0.21) | 0.08 (-0.08, 0.24) | -0.13 (-0.25, -0.01) | 0.18 (-0.11, 0.46) |
|  | Women | Minimally adjusted | 0.20 (0.13, 0.27)* | 0.05 (-0.16, 0.27) | 0.63 (0.17, 1.10) | 0.23 (0.01, 0.45) | -0.17 (-0.28, -0.06) | 0.14 (0.02, 0.27) | -0.08 (-0.23, 0.08) | -0.29 (-0.52, -0.06) |
|  |  | Adjusted | 0.13 (-0.04, 0.29) | -0.04 (-0.31, 0.23) | 0.39 (0.17, 0.62)* | 0.06 (-0.10, 0.21) | -0.11 (-0.21, 0.00) | 0.06 (-0.06, 0.17) | -0.05 (-0.18, 0.07) | -0.19 (-0.37, 0.00) |
| **IQ score (harmonized units)** | Both sexes | Minimally adjusted | 1.31 (1.00, 1.63)* | 0.02 (-0.76, 0.80) | 3.11 (2.20, 4.02)* | 1.03 (0.64, 1.42)* | -0.44 (-0.87, -0.01) | 0.31 (-0.14, 0.75) | 0.12 (-0.39, 0.64) | 0.17 (-1.47, 1.80) |
|  |  | Adjusted | 0.73 (0.35, 1.10)* | -0.61 (-1.77, 0.54) | 1.36 (0.64, 2.09)* | 0.24 (-0.36, 0.84) | -0.23 (-1.54, 1.08) | -0.18 (-0.98, 0.63) | 0.08 (-0.73, 0.89) | 0.40 (-0.61, 1.41) |
|  | Men | Minimally adjusted | 1.42 (0.94, 1.90)* | 0.53 (-2.00, 3.06) | 3.08 (1.78, 4.39)* | 0.90 (0.35, 1.45)* | -0.50 (-1.05, 0.06) | 0.26 (-0.29, 0.81) | 0.09 (-0.47, 0.65) | 1.05 (-0.08, 2.18) |
|  |  | Adjusted | 0.92 (0.48, 1.35)* | -0.87 (-3.38, 1.64) | 1.56 (0.59, 2.54) | 0.17 (-0.67, 1.00) | 0.42 (-1.35, 2.20) | -0.31 (-1.56, 0.95) | -0.14 (-1.12, 0.84) | 1.12 (-0.23, 2.47) |
|  | Women | Minimally adjusted | 1.24 (0.82, 1.65)* | -0.03 (-0.86, 0.79) | 3.14 (1.87, 4.41)* | 1.16 (0.62, 1.70)* | -0.34 (-1.03, 0.34) | 0.40 (-0.37, 1.17) | 0.30 (-1.00, 1.60) | -0.63 (-1.46, 0.21) |
|  |  | Adjusted | 0.53 (0.09, 0.97) | -0.54 (-1.84, 0.76) | 1.12 (0.04, 2.20) | 0.32 (-0.54, 1.17) | -0.91 (-2.73, 0.91) | -0.08 (-1.14, 0.97) | 0.56 (-0.87, 1.99) | 0.03 (-0.55, 0.61) |

^1^ Values are linear regression coefficients (*β*s and 95% CIs). IQ, intelligence quotient.

^2^ In minimally adjusted models, pooled models adjust for sex. In Guatemala analysis, we also controlled for year at birth and intervention group. In fully adjusted analyses, we controlled for maternal factors (height, age at childbirth, schooling), paternal schooling, birth order, and income/wealth quintiles. Additionally, we controlled for maternal skin color in both Brazil cohorts. **P* value < 0.001.

**Supplemental Table 4. Adjusted associations between conditional growth (birth length as anchor) in childhood and the number of years of school attainment in adulthood, by study site and sex^1^**

| **Study site** | **Sex** | **Birth length** | **Conditional birth weight** | **Conditional height at around 2 years of age** | **Conditional height in mid-childhood** | **Conditional height in adulthood** | **Conditional relative weight at around 2 years of age** | **Conditional relative weight in mid-childhood** | **Conditional relative weight in adulthood** | ***n^2^*** |
| --- | --- | --- | --- | --- | --- | --- | --- | --- | --- | --- |
| Brazil 1982 | Men | NA | NA | NA | NA | NA | NA | NA | NA | NA |
|  | Women | NA | NA | NA | NA | NA | NA | NA | NA | NA |
|  | Both sexes | NA | NA | NA | NA | NA | NA | NA | NA | NA |
| Brazil 1993 | Men | 0.16 (0.08, 0.25)* | 0.15 (-0.06, 0.37) | -0.01 (-0.24, 0.22) | 0.29 (0.03, 0.55) | -0.19 (-0.56, 0.17) | -0.11 (-0.35, 0.13) | -0.14 (-0.43, 0.14) | 0.00 (-0.26, 0.26) | 399 |
|  | Women | 0.05 (-0.02, 0.11) | 0.23 (0.00, 0.46) | 0.26 (0.07, 0.45) | 0.19 (-0.02, 0.40) | -0.11 (-0.33, 0.10) | 0.06 (-0.11, 0.23) | 0.00 (-0.21, 0.22) | 0.04 (-0.20, 0.27) | 478 |
|  | Both sexes | 0.10 (-0.01, 0.22) | 0.20 (0.04, 0.36) | 0.14 (-0.13, 0.40) | 0.24 (0.07, 0.40) | -0.15 (-0.35, 0.05) | -0.02 (-0.18, 0.15) | -0.06 (-0.24, 0.11) | 0.02 (-0.15, 0.20) | 877 |
| Guatemala | Men | 0.56 (0.20, 0.92) | 0.71 (-0.48, 1.91) | 0.63 (0.46, 0.81)* | 0.92 (0.51, 1.33)* | 0.28 (0.02, 0.55) | -0.04 (-0.90, 0.82) | -0.13 (-1.05, 0.79) | 0.61 (-0.26, 1.47) | 97 |
|  | Women | 0.03 (-0.37, 0.42) | -0.38 (-1.10, 0.33) | 1.22 (0.44, 2.01) | 0.60 (-0.06, 1.27) | 0.66 (-0.33, 1.64) | 0.52 (-0.35, 1.40) | 0.68 (-0.26, 1.63) | -1.18 (-1.94, -0.43) | 117 |
|  | Both sexes | 0.27 (-0.26, 0.79) | 0.09 (-0.97, 1.14) | 0.95 (0.35, 1.56) | 0.75 (0.34, 1.16)* | 0.49 (-0.06, 1.04) | 0.28 (-0.34, 0.91) | 0.33 (-0.47, 1.14) | -0.42 (-2.19, 1.35) | 214 |
| India | Men | 0.13 (-0.20, 0.45) | 0.64 (0.11, 1.17) | 0.62 (0.02, 1.22) | -0.16 (-0.81, 0.49) | 0.08 (-0.72, 0.87) | 0.63 (0.04, 1.23) | -0.21 (-0.68, 0.27) | 0.11 (-0.78, 1.00) | 275 |
|  | Women | 0.47 (0.17, 0.78) | -0.30 (-0.66, 0.06) | 0.69 (0.16, 1.23) | 0.17 (-0.27, 0.62) | -0.11 (-0.53, 0.31) | 0.41 (0.01, 0.81) | -0.36 (-0.73, 0.01) | -0.30 (-0.60, -0.01) | 242 |
|  | Both sexes | 0.29 (-0.05, 0.63) | 0.20 (-0.72, 1.12) | 0.65 (0.25, 1.06) | 0.00 (-0.41, 0.40) | -0.01 (-0.48, 0.46) | 0.53 (0.16, 0.90) | -0.28 (-0.59, 0.03) | -0.08 (-0.58, 0.41) | 517 |
| Philippines | Men | 0.13 (-0.05, 0.32) | -0.20 (-0.44, 0.04) | 0.16 (-0.16, 0.48) | 0.22 (-0.01, 0.45) | -0.18 (-0.44, 0.07) | -0.06 (-0.31, 0.19) | -0.20 (-0.50, 0.10) | 0.41 (0.11, 0.70) | 877 |
|  | Women | 0.07 (-0.14, 0.28) | 0.00 (-0.29, 0.28) | 0.40 (0.17, 0.63)* | 0.00 (-0.18, 0.19) | 0.03 (-0.19, 0.25) | 0.12 (-0.17, 0.40) | -0.21 (-0.47, 0.05) | -0.11 (-0.33, 0.12) | 783 |
|  | Both sexes | 0.10 (-0.04, 0.24) | -0.11 (-0.30, 0.08) | 0.27 (0.03, 0.51) | 0.12 (-0.10, 0.33) | -0.08 (-0.30, 0.13) | 0.02 (-0.17, 0.21) | -0.20 (-0.41, 0.00) | 0.17 (-0.34, 0.69) | 1660 |
| South Africa | Men | NA | NA | NA | NA | NA | NA | NA | NA | NA |
|  | Women | NA | NA | NA | NA | NA | NA | NA | NA | NA |
|  | Both sexes | NA | NA | NA | NA | NA | NA | NA | NA | NA |
| All sites^3^ | Men | 0.19 (0.07, 0.32) | 0.18 (-0.18, 0.54) | 0.28 (0.00, 0.56) | 0.22 (0.00, 0.45) | 0.03 (-0.14, 0.21) | 0.08 (-0.08, 0.24) | -0.13 (-0.25, -0.01) | 0.18 (-0.11, 0.46) | 3413 |
| I² statistic (%) |  | 36.9 | 72.4 | 88.3 | 76.9 | 53.1 | 53.5 | 0 | 72.5 |  |
| Cochran’s Q test *(P* value) |  | 0.191 | 0.012 | <0.001 | 0.001 | 0.059 | 0.056 | 0.81 | 0.003 |  |
| All sites | Women | 0.13 (-0.04, 0.29) | -0.04 (-0.31, 0.23) | 0.39 (0.17, 0.62)* | 0.06 (-0.10, 0.21) | -0.11 (-0.21, 0.00) | 0.06 (-0.06, 0.17) | -0.05 (-0.18, 0.07) | -0.19 (-0.37, 0.00) | 3458 |
| I² statistic (%) |  | 58.8 | 59.7 | 77.5 | 59.5 | 12.2 | 15.1 | 34.3 | 65.1 |  |
| Cochran’s Q test *(P* value) |  | 0.064 | 0.059 | <0.001 | 0.03 | 0.337 | 0.317 | 0.179 | 0.014 |  |
| All sites | Both sexes | 0.17 (0.07, 0.27)* | 0.04 (-0.18, 0.26) | 0.35 (0.17, 0.53)* | 0.12 (-0.04, 0.28) | -0.06 (-0.19, 0.07) | 0.07 (-0.03, 0.16) | -0.09 (-0.18, -0.01) | -0.02 (-0.38, 0.33) | 6871 |
| I² statistic (%) |  | 58.4 | 61.9 | 83.4 | 72.1 | 46.4 | 34.5 | 2.8 | 71.6 |  |
| Cochran’s Q test *(P* value) |  | 0.018 | 0.01 | <0.001 | <0.001 | 0.039 | 0.114 | 0.417 | <0.001 |  |

^1^ Values are linear regression coefficients (*β*s and 95% CIs). In adjusted analyses, we controlled for maternal factors (height, age at childbirth, schooling), birth order, and income/wealth quintiles. Additionally, we controlled for year at birth and intervention group in Guatemala, and for maternal skin color in both Brazil cohorts. **P* value < 0.001.

^2^ These models exclude cases with missing values.

^3^ These β coefficients are the same as presented in Supplemental Table 3 (adjusted models). The I² statistic and Cochran’s Q test were used to quantify between-cohort variation.

**Supplemental Table 5. Pooled adjusted associations between conditional growth in childhood (birth weight and birth length as anchor) and school attainment (binary variable), by sex^1^**

|  | **Sex** | **Models^2^** | **Birth weight** | **Birth length** | **Conditional birth weight** | **Conditional height at around 2 years of age** | **Conditional height in mid-childhood** | **Conditional height in adulthood** | **Conditional relative weight at around 2 years of age** | **Conditional relative weight in mid-childhood** | **Conditional relative weight in adulthood** |
| --- | --- | --- | --- | --- | --- | --- | --- | --- | --- | --- | --- |
| **School attainment (binary variable)^3^ Anchor birthweight** | Both sexes | Minimally adjusted | 1.07 (1.04, 1.09)* | .. | .. | 1.18 (1.12, 1.24)* | 1.06 (1.03, 1.09)* | 0.99 (0.94, 1.04) | 1.03 (0.99, 1.08) | 1.00 (0.98, 1.02) | 0.98 (0.88, 1.10) |
|  |  | Adjusted | 1.04 (1.02, 1.05)* | .. | .. | 1.07 (1.02, 1.11)* | 1.03 (0.99, 1.06) | 0.98 (0.93, 1.04) | 1.02 (0.97, 1.08) | 0.99 (0.94, 1.04) | 0.97 (0.94, 1.00) |
|  | Men | Minimally adjusted | 1.07 (1.03, 1.11)* | .. | .. | 1.20 (1.11, 1.29)* | 1.06 (1.02, 1.10)* | 1.02 (0.96, 1.07) | 1.06 (1.01, 1.11)* | 0.99 (0.95, 1.03) | 1.04 (0.98, 1.11) |
|  |  | Adjusted | 1.04 (1.01, 1.06)* | .. | .. | 1.04 (0.98, 1.10) | 1.02 (0.98, 1.06) | 1.01 (0.97, 1.05) | 1.06 (1.01, 1.11)* | 0.96 (0.93, 0.99)* | 1.01 (0.92, 1.11) |
|  | Women | Minimally adjusted | 1.07 (1.03, 1.10)* | .. | .. | 1.17 (1.09, 1.25)* | 1.06 (1.01, 1.12)* | 0.96 (0.93, 1.00)* | 1.01 (0.98, 1.04) | 1.01 (0.98, 1.04) | 0.93 (0.87, 0.99)* |
|  |  | Adjusted | 1.03 (1.01, 1.06)* | .. | .. | 1.08 (1.04, 1.12)* | 1.04 (0.98, 1.11) | 0.95 (0.92, 0.99)* | 1.00 (0.97, 1.02) | 1.01 (0.98, 1.04) | 0.96 (0.93, 1.00)* |
| **School attainment (binary variable) Anchor birth length** | Both sexes | Minimally adjusted | .. | 1.05 (1.03, 1.07)* | 1.03 (0.99, 1.07) | 1.18 (1.12, 1.24)* | 1.05 (1.02, 1.09) | 0.98 (0.94, 1.03) | 1.03 (0.98, 1.08) | 1.00 (0.97, 1.02) | 0.98 (0.88, 1.10) |
|  |  | Adjusted | .. | 1.04 (1.01, 1.07) | 1.01 (0.96, 1.07) | 1.06 (1.02, 1.10) | 1.04 (0.99, 1.08) | 0.99 (0.93, 1.05) | 1.02 (0.97, 1.08) | 0.99 (0.94, 1.04) | 0.99 (0.92, 1.07) |
|  | Men | Minimally adjusted | .. | 1.07 (1.04, 1.09)* | 1.03 (0.93, 1.13) | 1.19 (1.11, 1.28)* | 1.05 (1.00, 1.10) | 1.01 (0.96, 1.06) | 1.06 (1.01, 1.11)* | 0.99 (0.95, 1.03) | 1.04 (0.98, 1.11) |
|  |  | Adjusted | .. | 1.06 (1.02, 1.10) | 1.01 (0.92, 1.12) | 1.03 (0.96, 1.11) | 1.05 (0.98, 1.13) | 1.02 (0.99, 1.06) | 1.05 (1.01, 1.09) | 0.96 (0.93, 1.00) | 1.04 (0.96, 1.12) |
|  | Women | Minimally adjusted | .. | 1.04 (1.02, 1.07)* | 1.03 (0.98, 1.07) | 1.16 (1.08, 1.25)* | 1.06 (1.01, 1.10) | 0.96 (0.93, 0.99) | 1.01 (0.98, 1.04) | 1.01 (0.98, 1.04) | 0.93 (0.87, 0.99) |
|  |  | Adjusted |  | 1.02 (0.99, 1.06) | 1.01 (0.95, 1.08) | 1.07 (1.02, 1.12) | 1.03 (0.97, 1.08) | 0.96 (0.92, 1.00) | 1.00 (0.97, 1.03) | 1.01 (0.98, 1.04) | 0.96 (0.92, 1.00) |

^1^ Values are risk ratios (95% CIs). IQ, intelligence quotient.

^2^ In minimally adjusted models, pooled models adjust for sex. In Guatemala analysis, we also controlled for year at birth and intervention group. In fully adjusted analyses, we controlled for maternal factors (height, age at childbirth, schooling), paternal schooling, birth order, and income/wealth quintiles. Additionally, we controlled for maternal skin color in both Brazil cohorts. **P* value < 0.001.

^3^ We used site-specific thresholds relevant to secondary schooling (primary school in Guatemala) completion current at the time the cohort members were children (Brazil 1982: ≥ 12 years; Brazil 1993: ≥ 11 years; Guatemala: ≥ 6 years; India: > 12 years; The Philippines: ≥ 11 years; South Africa: ≥ 12 years).

**Supplemental Table 6. Adjusted associations between conditional growth in childhood (birth weight as anchor) and school attainment (binary variable)^1^, by study site and sex^2^**

| **Study site** | **Sex** | **Birth weight** | **Conditional height at around 2 years of age** | **Conditional height in mid-childhood** | **Conditional height in adulthood** | **Conditional relative weight at around 2 years of age** | **Conditional relative weight in mid-childhood** | **Conditional relative weight in adulthood** | ***n^3^*** |
| --- | --- | --- | --- | --- | --- | --- | --- | --- | --- |
| Brazil 1982 | Men | 1.04 (0.99, 1.10) | 1.10 (1.01, 1.19) | 0.99 (0.93, 1.06) | 0.98 (0.91, 1.06) | 1.08 (1.01, 1.15) | 0.98 (0.92, 1.04) | 0.97 (0.91, 1.02) | 1456 |
|  | Women | 1.06 (1.01, 1.11) | 1.10 (1.04, 1.17) | 1.00 (0.95, 1.05) | 0.97 (0.91, 1.03) | 0.98 (0.93, 1.04) | 1.02 (0.98, 1.07) | 0.90 (0.86, 0.95)* | 1500 |
|  | Both sexes | 1.05 (1.01, 1.09) | 1.10 (1.04, 1.16)* | 1.00 (0.96, 1.04) | 0.98 (0.93, 1.03) | 1.03 (0.94, 1.13) | 1.00 (0.96, 1.04) | 0.93 (0.87, 1.00) | 2956 |
| Brazil 1993 | Men | 1.05 (1.01, 1.08) | 0.91 (0.80, 1.04) | 1.08 (0.95, 1.23) | 0.97 (0.84, 1.13) | 1.05 (0.94, 1.16) | 1.00 (0.89, 1.13) | 0.96 (0.84, 1.11) | 403 |
|  | Women | 1.03 (1.01, 1.07) | 1.05 (0.97, 1.12) | 1.11 (1.02, 1.22) | 0.90 (0.84, 0.97) | 1.00 (0.93, 1.06) | 1.01 (0.94, 1.08) | 1.01 (0.94, 1.08) | 490 |
|  | Both sexes | 1.04 (1.02, 1.06)* | 0.98 (0.86, 1.13) | 1.10 (1.02, 1.19) | 0.93 (0.87, 1.01) | 1.02 (0.96, 1.08) | 1.01 (0.94, 1.07) | 0.99 (0.92, 1.07) | 893 |
| Guatemala | Men | 1.14 (1.00, 1.30) | 0.92 (0.77, 1.10) | 0.84 (0.68, 1.05) | 1.16 (0.96, 1.41) | 1.41 (0.92, 2.16) | 0.99 (0.71, 1.37) | 0.35 (0.18, 0.67) | 134 |
|  | Women | 1.09 (0.96, 1.22) | 1.03 (0.84, 1.27) | 1.45 (1.13, 1.87) | 0.96 (0.80, 1.15) | 1.12 (0.84, 1.49) | 1.14 (0.89, 1.47) | 0.96 (0.81, 1.14) | 148 |
|  | Both sexes | 1.11 (1.02, 1.21) | 0.98 (0.85, 1.12) | 1.12 (0.66, 1.91) | 1.05 (0.87, 1.27) | 1.24 (0.97, 1.59) | 1.07 (0.87, 1.31) | 0.62 (0.23, 1.66) | 282 |
| India | Men | 1.09 (0.95, 1.25) | 1.06 (0.91, 1.23) | 0.97 (0.86, 1.10) | 1.00 (0.90, 1.11) | 1.10 (0.97, 1.25) | 0.97 (0.86, 1.10) | 0.98 (0.88, 1.10) | 280 |
|  | Women | 1.01 (0.94, 1.08) | 1.20 (1.06, 1.35) | 1.09 (1.00, 1.19) | 0.96 (0.90, 1.04) | 1.04 (0.96, 1.12) | 1.03 (0.87, 1.21) | 0.97 (0.89, 1.05) | 243 |
|  | Both sexes | 1.05 (0.97, 1.14) | 1.12 (0.99, 1.27) | 1.03 (0.91, 1.15) | 0.98 (0.92, 1.05) | 1.07 (0.99, 1.15) | 1.00 (0.90, 1.11) | 0.97 (0.91, 1.05) | 523 |
| Philippines | Men | 0.99 (0.94, 1.05) | 1.09 (1.01, 1.18) | 1.05 (0.99, 1.12) | 1.00 (0.94, 1.07) | 0.99 (0.94, 1.05) | 0.95 (0.90, 1.00) | 1.07 (1.00, 1.14) | 877 |
|  | Women | 1.01 (0.95, 1.08) | 1.10 (1.03, 1.18) | 1.01 (0.96, 1.07) | 0.99 (0.93, 1.06) | 1.00 (0.95, 1.06) | 0.99 (0.94, 1.04) | 0.99 (0.94, 1.04) | 783 |
|  | Both sexes | 1.00 (0.96, 1.05) | 1.10 (1.04, 1.16)* | 1.03 (0.99, 1.08) | 1.00 (0.95, 1.05) | 1.00 (0.96, 1.04) | 0.97 (0.93, 1.01) | 1.03 (0.95, 1.12) | 1660 |
| South Africa | Men | 1.02 (0.95, 1.09) | 1.03 (0.93, 1.16) | 1.04 (0.96, 1.13) | 1.09 (0.98, 1.21) | 1.09 (1.00, 1.18) | 0.93 (0.86, 1.02) | 1.19 (1.09, 1.30)* | 309 |
|  | Women | 1.02 (0.95, 1.09) | 1.03 (0.95, 1.11) | 0.94 (0.87, 1.01) | 0.88 (0.77, 1.01) | 0.97 (0.90, 1.05) | 1.03 (0.95, 1.12) | 0.96 (0.91, 1.02) | 338 |
|  | Both sexes | 1.02 (0.97, 1.07) | 1.03 (0.96, 1.10) | 0.98 (0.89, 1.09) | 0.98 (0.79, 1.20) | 1.02 (0.92, 1.14) | 0.98 (0.89, 1.08) | 1.07 (0.87, 1.31) | 647 |
| All sites^4^ | Men | 1.04 (1.01, 1.06) | 1.04 (0.98, 1.10) | 1.02 (0.98, 1.06) | 1.01 (0.97, 1.05) | 1.06 (1.01, 1.11) | 0.96 (0.93, 0.99) | 1.01 (0.92, 1.11) | 3459 |
| I² statistic (%) |  | 3.6 | 41.4 | 13.3 | 0 | 39.7 | 0 | 82 |  |
| Cochran’s Q test *(P* value) |  | 0.393 | 0.129 | 0.329 | 0.432 | 0.141 | 0.921 | <0.001 |  |
| All sites | Women | 1.03 (1.01, 1.06)* | 1.08 (1.04, 1.12)* | 1.04 (0.98, 1.11) | 0.95 (0.92, 0.99) | 1.00 (0.97, 1.02) | 1.01 (0.98, 1.04) | 0.96 (0.93, 1.00) | 3502 |
| I² statistic (%) |  | 0 | 17.6 | 74.2 | 11.2 | 0 | 0 | 42.8 |  |
| Cochran’s Q test *(P* value) |  | 0.76 | 0.3 | 0.002 | 0.344 | 0.807 | 0.827 | 0.12 |  |
| All sites | Both sexes | 1.04 (1.02, 1.05)* | 1.07 (1.02, 1.11) | 1.03 (0.99, 1.06) | 0.98 (0.93, 1.04) | 1.02 (0.97, 1.08) | 0.99 (0.94, 1.04) | 0.97 (0.94, 1.00) | 6961 |
| I² statistic (%) |  | 0 | 30.3 | 56.2 | 29.5 | 29.4 | 0 | 76 |  |
| Cochran’s Q test *(P* value) |  | 0.725 | 0.149 | 0.009 | 0.156 | 0.157 | 0.648 | <0.001 |  |

^1^ We used site-specific thresholds relevant to secondary schooling (primary school in Guatemala) completion current at the time the cohort members were children (Brazil 1982: ≥ 12 years; Brazil 1993: ≥ 11 years; Guatemala: ≥ 6 years; India: > 12 years; The Philippines: ≥ 11 years; South Africa: ≥ 12 years).

^2^ Values are risk ratios (95% CIs). In adjusted analyses, we controlled for maternal factors (height, age at childbirth, schooling), birth order, and income/wealth quintiles. Additionally, we controlled for year at birth and intervention group in Guatemala, and for maternal skin color in both Brazil cohorts. **P* value < 0.001.

^3^ These models exclude cases with missing values.

^4^ These prevalence ratios are the same as presented in Supplemental Table 5 (adjusted models). The I² statistic and Cochran’s Q test were used to quantify between-cohort variation.

**Supplemental Table 7. Adjusted associations between conditional growth in childhood (birth length as anchor) and school attainment (binary variable)^1^, by study site and sex^2^**

| **Study site** | **Sex** | **Birth length** | **Conditional birth weight** | **Conditional height at around 2 years of age** | **Conditional height in mid-childhood** | **Conditional height in adulthood** | **Conditional relative weight at around 2 years of age** | **Conditional relative weight in mid-childhood** | **Conditional relative weight in adulthood** | ***n^3^*** |
| --- | --- | --- | --- | --- | --- | --- | --- | --- | --- | --- |
| Brazil 1982 | Men | NA | NA | NA | NA | NA | NA | NA | NA | NA |
|  | Women | NA | NA | NA | NA | NA | NA | NA | NA | NA |
|  | Both sexes | NA | NA | NA | NA | NA | NA | NA | NA | NA |
| Brazil 1993 | Men | 1.05 (1.02, 1.08)* | 1.01 (0.91, 1.13) | 0.92 (0.80, 1.05) | 1.05 (0.92, 1.21) | 0.97 (0.83, 1.12) | 1.05 (0.94, 1.17) | 1.01 (0.90, 1.13) | 0.97 (0.84, 1.12) | 399 |
|  | Women | 1.01 (0.98, 1.03) | 1.09 (1.01, 1.19) | 1.07 (0.99, 1.15) | 1.10 (1.01, 1.20) | 0.91 (0.85, 0.98) | 1.01 (0.93, 1.09) | 1.01 (0.95, 1.09) | 1.01 (0.95, 1.09) | 478 |
|  | Both sexes | 1.03 (0.99, 1.07) | 1.06 (0.98, 1.14) | 1.00 (0.86, 1.15) | 1.08 (1.00, 1.17) | 0.93 (0.86, 1.01) | 1.03 (0.96, 1.10) | 1.01 (0.95, 1.08) | 0.99 (0.92, 1.07) | 877 |
| Guatemala | Men | 1.20 (1.05, 1.38) | 1.40 (0.92, 2.13) | 0.74 (0.54, 1.01) | 1.46 (1.21, 1.77)* | 1.06 (1.00, 1.13) | 1.08 (0.65, 1.81) | 0.73 (0.30, 1.81) | 1.43 (0.51, 4.01) | 97 |
|  | Women | 1.00 (0.86, 1.18) | 0.98 (0.70, 1.36) | 0.96 (0.87, 1.06) | 1.32 (0.94, 1.87) | 1.07 (0.94, 1.23) | 1.27 (0.91, 1.77) | 1.19 (0.84, 1.69) | 0.82 (0.60, 1.10) | 117 |
|  | Both sexes | 1.09 (0.91, 1.30) | 1.14 (0.81, 1.61) | 0.85 (0.66, 1.09) | 1.38 (1.12, 1.70) | 1.07 (0.99, 1.16) | 1.19 (0.89, 1.59) | 0.97 (0.63, 1.50) | 1.04 (0.64, 1.69) | 214 |
| India | Men | 1.07 (0.99, 1.16) | 1.09 (0.94, 1.28) | 1.14 (0.97, 1.34) | 0.95 (0.85, 1.08) | 0.99 (0.88, 1.12) | 1.11 (0.97, 1.26) | 0.99 (0.87, 1.11) | 0.99 (0.88, 1.10) | 275 |
|  | Women | 1.09 (1.02, 1.17) | 0.95 (0.88, 1.02) | 1.15 (1.04, 1.27) | 1.08 (0.99, 1.19) | 0.96 (0.89, 1.03) | 1.04 (0.95, 1.13) | 1.03 (0.87, 1.22) | 0.96 (0.89, 1.04) | 242 |
|  | Both sexes | 1.08 (1.03, 1.14) | 1.02 (0.88, 1.18) | 1.14 (1.04, 1.26) | 1.01 (0.89, 1.15) | 0.98 (0.91, 1.05) | 1.07 (0.99, 1.16) | 1.01 (0.91, 1.11) | 0.97 (0.91, 1.04) | 517 |
| Philippines | Men | 1.03 (0.97, 1.08) | 0.93 (0.88, 0.99) | 1.06 (0.98, 1.15) | 1.05 (0.98, 1.12) | 1.00 (0.93, 1.07) | 0.99 (0.94, 1.05) | 0.95 (0.90, 1.00) | 1.07 (1.00, 1.14) | 877 |
|  | Women | 1.00 (0.95, 1.05) | 1.01 (0.96, 1.06) | 1.10 (1.03, 1.18) | 1.02 (0.97, 1.07) | 1.00 (0.93, 1.07) | 1.00 (0.95, 1.06) | 0.99 (0.94, 1.04) | 0.99 (0.93, 1.04) | 783 |
|  | Both sexes | 1.01 (0.98, 1.05) | 0.97 (0.90, 1.05) | 1.08 (1.02, 1.14) | 1.03 (0.99, 1.08) | 1.00 (0.95, 1.05) | 1.00 (0.96, 1.04) | 0.97 (0.93, 1.00) | 1.03 (0.95, 1.12) | 1660 |
| South Africa | Men | NA | NA | NA | NA | NA | NA | NA | NA | NA |
|  | Women | NA | NA | NA | NA | NA | NA | NA | NA | NA |
|  | Both sexes | NA | NA | NA | NA | NA | NA | NA | NA | NA |
| All sites^4^ | Men | 1.06 (1.02, 1.10) | 1.01 (0.92, 1.12) | 1.03 (0.96, 1.11) | 1.05 (0.98, 1.13) | 1.02 (0.99, 1.06) | 1.05 (1.01, 1.09) | 0.96 (0.93, 1.00) | 1.04 (0.96, 1.12) | 3413 |
|  |  | (1.02, 1.10) | (0.92, 1.12) | (0.96, 1.11) | (0.98, 1.13) | (0.99, 1.06) | (1.01, 1.09) | (0.93, 1.00) | (0.96, 1.12) |  |
| I² statistic (%) |  | 35.6 | 60.9 | 52.8 | 68.1 | 6.2 | 20.2 | 0 | 71.3 |  |
| Cochran’s Q test (P value) |  | 0.199 | 0.053 | 0.06 | 0.008 | 0.377 | 0.281 | 0.835 | 0.004 |  |
| All sites | Women | 1.02 (0.99, 1.06) | 1.01 (0.95, 1.08) | 1.07 (1.02, 1.12) | 1.03 (0.97, 1.08) | 0.96 (0.92, 1.00) | 1.00 (0.97, 1.03) | 1.01 (0.98, 1.04) | 0.96 (0.92, 1.00) | 3458 |
| I² statistic (%) |  | 44.7 | 53.3 | 45.3 | 61.7 | 36.8 | 0 | 0 | 50.5 |  |
| Cochran’s Q test (P value) |  | 0.143 | 0.093 | 0.104 | 0.023 | 0.161 | 0.591 | 0.822 | 0.072 |  |
| All sites | Both sexes | 1.04 (1.01, 1.07) | 1.01 (0.96, 1.07) | 1.06 (1.02, 1.10) | 1.04 (0.99, 1.08) | 0.99 (0.93, 1.05) | 1.02 (0.97, 1.08) | 0.99 (0.94, 1.04) | 0.99 (0.92, 1.07) | 6871 |
| I² statistic (%) |  | 51.7 | 56.1 | 46.3 | 62.4 | 45 | 23.5 | 0 | 71.5 |  |
| Cochran’s Q test (P value) |  | 0.043 | 0.026 | 0.039 | 0.002 | 0.045 | 0.213 | 0.61 | <0.001 |  |

^1^ We used site-specific thresholds relevant to secondary schooling (primary school in Guatemala) completion current at the time the cohort members were children (Brazil 1982: ≥ 12 years; Brazil 1993: ≥ 11 years; Guatemala: ≥ 6 years; India: > 12 years; The Philippines: ≥ 11 years; South Africa: ≥ 12 years).

^2^ Values are risk ratios (95% CIs). In adjusted analyses, we controlled for maternal factors (height, age at childbirth, schooling), birth order, and income/wealth quintiles. Additionally, we controlled for year at birth and intervention group in Guatemala, and for maternal skin color in both Brazil cohorts. NA, not available data (length at birth was not available in Brazil 1982 and South Africa). **P* value < 0.001.

^3^ These models exclude cases with missing values.

^4^ These prevalence ratios are the same as presented in Supplemental Table 5 (adjusted models). The I² statistic and Cochran’s Q test were used to quantify between-cohort variation.

**Supplemental Table 8. Adjusted associations between conditional growth in childhood (birth length as anchor) and IQ in adulthood using, by study site and sex^1^**

| **Study site** | **Sex** | **Birth length** | **Conditional birth weight** | **Conditional height at around 2 years of age** | **Conditional height in mid-childhood** | **Conditional height in adulthood** | **Conditional relative weight at around 2 years of age** | **Conditional relative weight in mid-childhood** | **Conditional relative weight in adulthood** | ***n^2^*** |
| --- | --- | --- | --- | --- | --- | --- | --- | --- | --- | --- |
| Brazil 1982 | Men | NA | NA | NA | NA | NA | NA | NA | NA | NA |
|  | Women | NA | NA | NA | NA | NA | NA | NA | NA | NA |
|  | Both sexes | NA | NA | NA | NA | NA | NA | NA | NA | NA |
| Brazil 1993 | Men | 0.92 (0.43, 1.41)* | 1.20 (-0.31, 2.71) | 2.55 (0.63, 4.47) | 1.50 (-0.36, 3.37) | 0.37 (-1.15, 1.88) | 0.47 (-1.91, 2.85) | -0.73 (-2.37, 0.90) | -0.88 (-2.84, 1.08) | 378 |
|  | Women | 0.49 (-0.01, 1.00) | -0.06 (-1.43, 1.31) | 0.69 (-0.73, 2.11) | 1.21 (-0.30, 2.72) | 0.13 (-1.42, 1.68) | 1.36 (0.06, 2.66) | 0.35 (-1.30, 2.00) | 0.10 (-1.41, 1.60) | 456 |
|  | Both sexes | 0.70 (0.28, 1.12)* | 0.51 (-0.72, 1.74) | 1.53 (-0.28, 3.34) | 1.34 (0.16, 2.53) | 0.24 (-0.85, 1.33) | 0.96 (-0.33, 2.25) | -0.14 (-1.31, 1.03) | -0.35 (-1.56, 0.86) | 834 |
| Guatemala | Men | 1.21 (-1.12, 3.53) | -3.61 (-8.31, 1.10) | 3.13 (-0.33, 6.58) | 2.78 (-1.40, 6.96) | 5.19 (2.64, 7.73)* | -5.18 (-8.29, -2.07) | 5.00 (0.67, 9.32) | 8.49 (1.20, 15.79) | 94 |
|  | Women | 0.66 (-0.64, 1.96) | -2.72 (-5.35, -0.09) | -5.60 (-11.70, 0.50) | 1.68 (-0.97, 4.34) | -7.30 (-11.59, -3.01)* | -2.20 (-4.92, 0.52) | 3.90 (0.12, 7.67) | 1.66 (-1.81, 5.14) | 117 |
|  | Both sexes | 0.91 (-0.36, 2.17) | -3.09 (-5.60, -0.59) | -1.71 (-10.39, 6.97) | 2.17 (-0.20, 4.55) | -1.74 (-14.07, 10.60) | -3.46 (-6.38, -0.54) | 4.36 (1.52, 7.21) | 4.54 (-1.98, 11.07) | 211 |
| India | Men | NA | NA | NA | NA | NA | NA | NA | NA | NA |
|  | Women | NA | NA | NA | NA | NA | NA | NA | NA | NA |
|  | Both sexes | NA | NA | NA | NA | NA | NA | NA | NA | NA |
| Philippines | Men | 0.84 (-0.21, 1.88) | -1.69 (-2.86, -0.52) | 0.04 (-1.30, 1.38) | 0.23 (-0.98, 1.43) | -2.06 (-3.24, -0.88)* | -0.28 (-1.49, 0.94) | 0.31 (-0.96, 1.57) | 1.34 (-0.25, 2.93) | 672 |
|  | Women | 0.67 (-0.65, 1.99) | -0.02 (-1.49, 1.44) | 2.47 (1.19, 3.75)* | -1.17 (-2.50, 0.15) | 1.99 (0.42, 3.56) | 0.67 (-0.74, 2.08) | -1.46 (-2.84, -0.07) | -0.09 (-1.54, 1.36) | 576 |
|  | Both sexes | 0.76 (-0.07, 1.59) | -0.93 (-2.56, 0.69) | 1.16 (-1.23, 3.55) | -0.42 (-1.79, 0.95) | -0.19 (-4.17, 3.78) | 0.15 (-0.77, 1.07) | -0.49 (-2.22, 1.24) | 0.69 (-0.72, 2.10) | 1248 |
| South Africa | Men | NA | NA | NA | NA | NA | NA | NA | NA | NA |
|  | Women | NA | NA | NA | NA | NA | NA | NA | NA | NA |
|  | Both sexes | NA | NA | NA | NA | NA | NA | NA | NA | NA |
| All sites^3^ | Men | 0.92 (0.48, 1.35)* | -0.87 (-3.38, 1.64) | 1.56 (0.59, 2.54) | 0.17 (-0.67, 1.00) | 0.42 (-1.35, 2.20) | -0.31 (-1.56, 0.95) | -0.14 (-1.12, 0.84) | 1.12 (-0.23, 2.47) | 2898 |
| I² statistic (%) |  | 0 | 80.5 | 47.1 | 33.1 | 85.9 | 69.6 | 56.6 | 67.9 |  |
| Cochran’s Q test (P value) |  | 0.959 | 0.006 | 0.109 | 0.201 | <0.001 | 0.011 | 0.056 | 0.014 |  |
| All sites | Women | 0.53 (0.09, 0.97) | -0.54 (-1.84, 0.76) | 1.12 (0.04, 2.20) | 0.32 (-0.54, 1.17) | -0.91 (-2.73, 0.91) | -0.08 (-1.14, 0.97) | 0.56 (-0.87, 1.99) | 0.03 (-0.55, 0.61) | 2980 |
| I² statistic (%) |  | 0 | 42.4 | 56.7 | 44.8 | 83.6 | 62.4 | 76.1 | 0 |  |
| Cochran’s Q test (P value) |  | 0.951 | 0.176 | 0.056 | 0.123 | <0.001 | 0.031 | 0.002 | 0.922 |  |
| All sites | Both sexes | 0.73 (0.35, 1.10)* | -0.61 (-1.77, 0.54) | 1.36 (0.64, 2.09)* | 0.24 (-0.36, 0.84) | -0.23 (-1.54, 1.08) | -0.18 (-0.98, 0.63) | 0.08 (-0.73, 0.89) | 0.40 (-0.61, 1.41) | 5878 |
| I² statistic (%) |  | 0 | 64.3 | 48.2 | 33.5 | 82.9 | 62.3 | 65.5 | 45.6 |  |
| Cochran’s Q test (P value) |  | 0.893 | 0.016 | 0.043 | 0.14 | <0.001 | 0.005 | 0.002 | 0.056 |  |

^1^ Values are adjusted linear regression coefficients (*β*s and 95% CIs). IQ, Intelligence quotient; NA, not available data (length at birth not available in Brazil 1982 and South Africa, and IQ not available in India). In adjusted analyses, we controlled for maternal factors (height, age at childbirth, schooling), birth order, and income/wealth quintiles. Additionally, we controlled for year at birth and intervention group in Guatemala, and for maternal skin color in both Brazil cohorts. **P* value < 0.001.

^2^ These models exclude cases with missing values.

^3^ These β coefficients are the same as presented in Supplemental Table 3 (adjusted models). The I² statistic and Cochran’s Q test were used to quantify between-cohort variation.
